# Natural Resistance to and Attraction of the Cabbage Root Fly () in Oilseed Rape Accessions

**DOI:** 10.1021/acs.jafc.5c03948

**Published:** 2025-07-02

**Authors:** Rebekka Sontowski, Amine Abbadi, Hannah Koller, Steffen Rietz, Andreas Schedl, Alexander Weinhold, Nicole M. van Dam

**Affiliations:** 1 Institute of Biodiversity, Ecology, and Evolution, Friedrich Schiller University Jena, Dornburger Str. 143, Jena 07743,Germany; 2 Leibnitz Institute of Vegetable and Ornamental Crops (IGZ), Theodor-Echtermeyer-Weg 1, Großbeeren 14979, Germany; 3 German Centre for Integrative Biodiversity Research (iDiv) Halle-Jena-Leipzig, Puschstr. 4, Leipzig 04103, Germany; 4 NPZ Innovation GmbH, Hohenlieth-Hof 1, Holtsee 24363, Germany; 5 DBFZ Deutsches Biomasseforschungszentrum gemeinnützige GmbH, Torgauer Straße 116, Leipzig 04347, Germany

**Keywords:** *Brassica napus*, plant−insect interaction, pest management, belowground herbivore, root metabolome, plant volatile organic compounds

## Abstract

Winter oilseed rape (OSR, ) is an important crop and frequently attacked by the cabbage root fly larvae (). They damage the taproot, cause plant death, and negatively impact yield. Options to control with synthetic pesticides are limited, and no tolerance in OSR has been described. We aim to identify natural resistance in OSR accessions to as a starting point for plant breeding. We tested OSR accessions for differences in larval feeding damage, larval performance, female attraction, and oviposition preference. Using a subset of the most and least resistant accessions, we analyzed volatile profiles and leaf traits and assessed root metabolic fingerprints. Resistance to larval damage was related to lower aliphatic glucosinolates and higher lignin-related compound levels. Oviposition preference was linked to higher trichome density. Our results suggest breeding efforts should focus on increasing lignin and decreasing specific glucosinolates in OSR roots.

## Graphical abstract

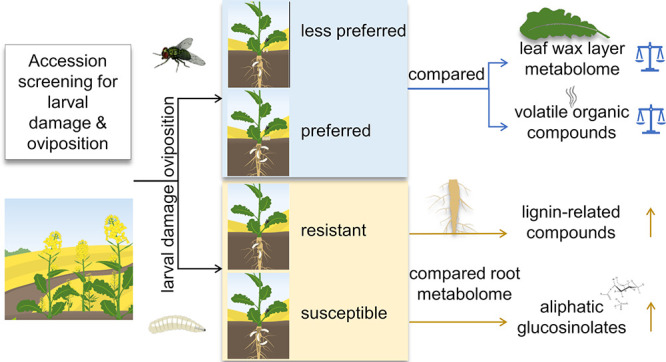

## Introduction

Winter oilseed rape (OSR, *Brassica napus* L., Brassicaceae) is a widely grown crop in Europe, used for the production of vegetable oil, fuel, and fodder for livestock farming. With 11.6 million tons of OSR seeds produced yearly, Europe is one of the most important producers worldwide (2022, www.fao.org/faostat). In recent decades, breeding programs have mainly focused on increasing yield, oil quality and resistance to diseases. However, the expansion of OSR cropping was accompanied by the spread of various pests and pathogens. In total, 15 diseases and 35 insect species, nematodes, slugs and snails are described to attack OSR.^1^ Unfortunately, pest and disease control have become increasingly difficult. Recently, the European Union adopted the EU Biodiversity Strategy. It states that the use and risk of synthetic chemical pesticides should be reduced by 50% by 2030 (EU, 2020). Several synthetic chemical pesticides have already been banned due to concerns about their negative effects on human health and ecosystems, for instance, on nontarget pollinators and soil microorganisms.^2,3^ In addition, pests are becoming less sensitive to chemical control due to repeated application of remaining active agents. This urges the development of novel and integrated pest management strategies to control OSR pests effectively. In particular, pest management focusing on brassicaceous plants, including OSR, has recently been intensively studied.^4–6^

Like most other brassicaceous plant species, OSR produces glucosinolates (GSLs) as a chemical defense. The defense consists of two components, the GSLs and the enzyme myrosinase, which are stored in specific cells. When herbivores damage the plant, GSLs and myrosinases mix, producing toxic and noxious compounds, such as isothiocyanates and nitriles.^7^ Despite this so-called mustard-oil bomb, OSR plants are attacked by various insect herbivores. These herbivores possess counter-adaptations that help to neutralize or detoxify the GSL system.^8–10^ Some of these species even specialize in brassicaceous plants and Brassicaceae-specific chemical cues stimulate their feeding or oviposition behavior.^11,12^ Common specialized belowground pests in OSR are the larvae of the cabbage root fly *Delia radicum* L. (Anthomyiidae).^13,14^ Due to recently unveiled detoxification mechanisms, *D. radicum* larvae feed on the taproot, even though the roots of *Brassica* species are known to produce high levels of GSLs and isothiocyanates.^15–17^ As the resulting plant lesion is underneath the soil, the consequences are usually discovered too late to prevent damage. Moreover, the effective pesticides used to combat cabbage root fly are either banned or increase the possibility of developing insect resistance.^18^ Therefore, it is crucial to identify novel strategies to reduce *D. radicum* damage in OSR crops.

In contrast to the larvae, adult flies do not directly harm the plant.^19^ However, the females select the host plant for their offspring by depositing eggs on the soil surface near the stem of brassicaceous plants.^20^ Female flies choose a host plant based on chemical and physical cues to select the best host plant for their offspring. Following the ‘mother knows best’ hypothesis, *D. radicum* females show a sequence of behaviors to select a ‘good’ plant for their offspring. After locating and identifying a cruciferous plant, females land on the leaves and assess the leaf quality by walking on the leaf surface.^20–22^ During their walk, females probe the chemical composition of the leaf surface. Specific chemical compounds on the leaf surface activate particular receptors on the tarsal sensilla and stimulate oviposition behavior.^22^ In the next step, they migrate up and down the stem, circling the root shoot interface and testing the soil surface conditions, followed by a decision on egg deposition.^23^ Oviposition preference depends on various plant conditions, for instance, leaf shape, size, color and plant volatile organic compound (VOC) profile.^24–27^ In addition, the physical and chemical properties of the cuticular wax layer inform insects about the plant’s quality, thus contributing to the acceptance or rejection of the host plant for oviposition.^28^

In order to identify novel natural resources of resistance to cabbage root fly in OSR, we focused on the natural resistance and attractiveness of intraspecific variation among OSR accessions to the specialist *D. radium*. Using a panel of 20 OSR accessions, selected for having either high, intermediate or low GSL levels from a larger set of 45 accessions, we experimentally assessed differences in natural resistance to the *D. radicum* larvae and in attraction of *D. radicum* females. Using a subset of the most and least attractive accessions, we performed LC-MS based analyses of root tissues and GC-MS based analyses of leaf cuticular composition and volatile bouquets emitted by the whole plant. This allowed us to link larval performance to the root metabolome and oviposition preference to emitted plant volatiles, cuticular wax layer metabolome of leaves and trichome density. Finally, we correlated female selection of OSR accessions with larval preference. These findings increase our understanding of the natural resistance in OSR accessions to the specialist *D. radicum* at both agriculturally relevant life stages.

## Methods and Materials

### Accession Selection

A total of 45 OSR accessions were selected by NPZ Innovation GmbH, Holtsee, Germany. From each accession, ten plants were grown as described in Supporting Information S1. After 6 weeks, the whole roots were harvested, washed with tap water, flash-frozen in liquid nitrogen, freeze-dried and ground (mixer mill Retsch MM400, 30 Hz, for 5 min). We extracted and measured the total GSLs of the roots according to Grosser and van Dam.^29^ In brief, GSLs were desulfated and analyzed by reversed-phase ultrahigh performance liquid chromatography (UHPLC) equipped with a photodiode array detector (PDA, Thermo Scientific Ultimate 3000 series). Based on the GSL profile of the root, 20 OSR accessions were selected that contained high levels of aliphatic, indole or low GSLs, a mixture of GSLs or were outliers (Figure 1, Table S1).

**Figure 1:**
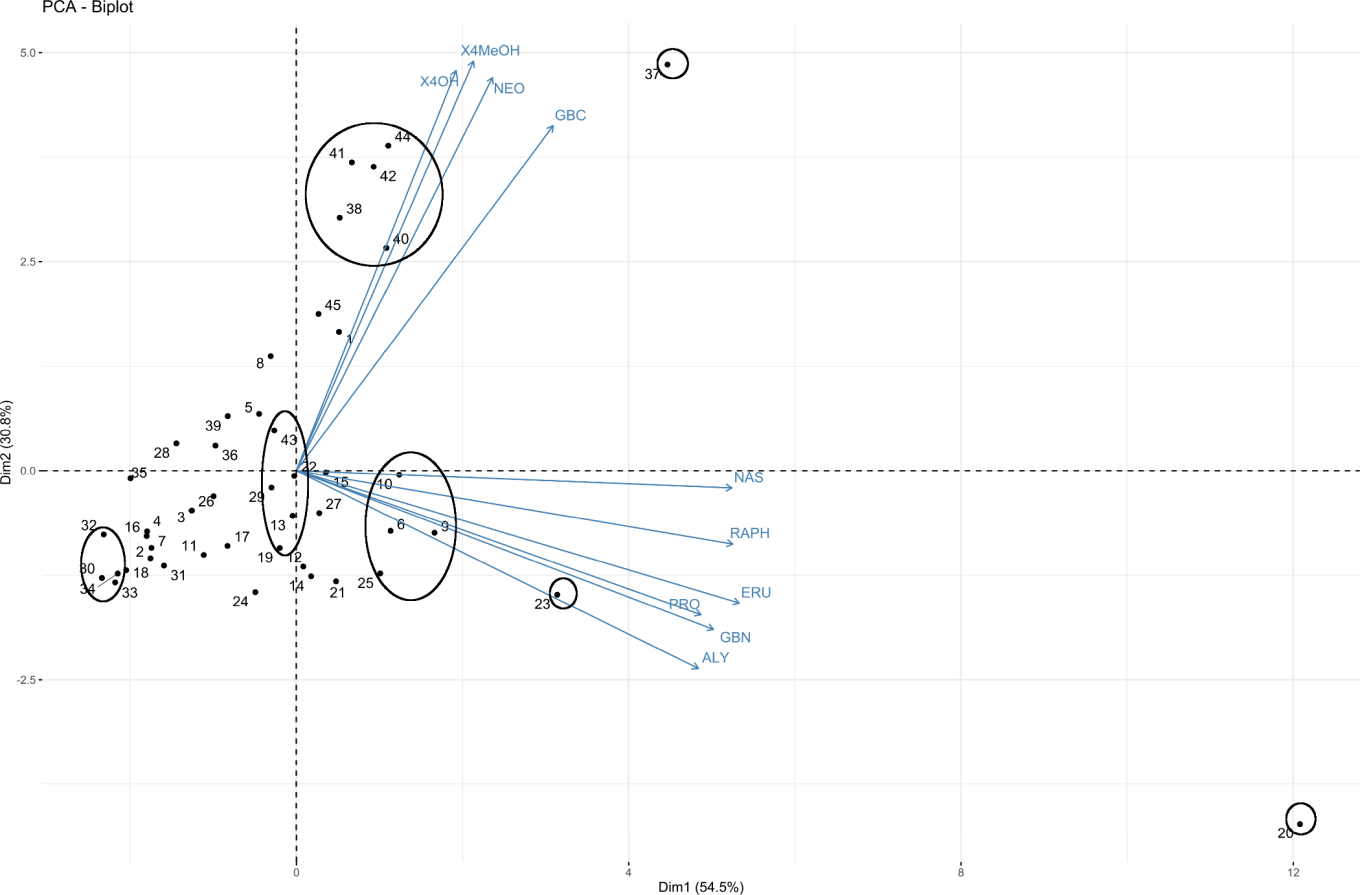
PCA biplot of glucosinolate profiles in the roots of 45 *Brassica napus* accessions. The number represents the *B. napus* accession. Blue color presents specific glucosinolates driving the separation. NAS: Gluconasturtiin, RAPH: Glucoraphanin, ERU: Glucoerucin, GBN: Glucobrassicanapin, PRO: Progoitrin, ALY: Glucoalyssin, NEO: Gluconeobrassicin, GBC: Glucobrassicin, X4MeOH: 4-Methoxyglucobrassicin, X4OH: 4-Hydroxyglucobrassicin.

#### Larval Resistance in the Roots

##### Semifield Experiment

The selected 20 OSR accessions were planted on the 23rd of August 2021 in the greenhouse to foster homogeneous plant development. Of each accession, 20 kernels were distributed on three multipot trays filled with peat-based soil and grown at 18 °C/22 °C (night/day) and 16 h light. Three weeks after sowing, trays with grown plants were transferred outside the greenhouse, surrounded by lawn. At this time of the year *D. radicum* infests naturally. Of the 20 plants per accession, 10 were infected with ten eggs of *D. radicum* each (provided by iDiv, Supporting Information S1). The remaining ten plants were exposed to natural infestation only. On the 11th of November, all plants were extracted from the soil to score larval damage at the roots. Scoring considered feeding damage and root development and ranked from scoring note 1 (no damage) to scoring note 4 (severe feeding damage with root system deterioration).

##### Greenhouse Experiment

We repeated the field experiment in the greenhouse with the same 20 OSR accessions as above. Between 17 and 20 plants were grown from each accession, except for accession NP-38 for which we had only 12 plants due to a low germination rate. This resulted in a total of 354 plants (more details on the growing conditions are described in Supporting Information S1). After 24 days, half of the plants were manually infested with ten eggs of *D. radicum*, which were laid within 24 h before they were used in the experiment. After 2 weeks (BBHC scale 13 ^30^), the taproot and lateral roots were harvested by removing roots from the sand, cutting off the aerial parts using garden clippers, rinsing them with tap water, and drying them with paper towels. Root damage was assessed on a scale from 0 (no damage) to 4 (severing of the roots, according to Danner et al.^31^). The harvested roots were frozen in liquid nitrogen, freeze-dried and weighed. The root dry mass served as a proxy for plant performance. The dried taproots were ground to a fine powder using an MM 400 grinding mill (Retsch, Haan, Germany) at 30 Hz for 1 min. The larvae and pupae of each plant were collected by flooding the sand with tap water. The collected larvae and pupae were counted and weighed.

##### Root Metabolic Profile of Selected Resistant and Susceptible Accessions

To correlate the root metabolome with larval feeding damage and larval performance, we measured the root metabolomic profile of the resistant and susceptible OSR accessions. First, we weighed 20 ± 1 mg of freeze-dried root material per plant. The metabolites were extracted in a methanol–water mixture as Weinhold et al. described.^32^ The buffer volume was adjusted according to the sample weight to obtain 50 μL buffer per mg sample. We measured the metabolites with an LC-ESI-Q-ToF-MS. In brief, chromatographic separations were performed on an UltiMate 3000 Ultra-High Pressure Liquid Chromatography system (UHPLC, Thermo Scientific) equipped with an Acclaim Rapid Separation Liquid Chromatography (RSLC) 120 column (150 × 2.1 mm, particle size 2.2 μm, Thermo Fischer Scientific). Eluted compounds were detected from *m*/*z* 90 to 1600 at a spectra rate of 5 Hz (accession spectra only), using an ESI-UHR-Q-ToF-MS (maXis impact, Bruker Daltonics) equipped with an Apollo II electrospray ion source. The LC-MS data, measured in positive and negative mode, were processed separately using MZmine version 3.9.0.^33^ Annotated compounds were matched against an in-house library of analytical standards of plant metabolites (Döll, unpublished), NIST17, and GNPS^34^ based on mass, retention time, spectrum and spectral similarity of MS2 spectra. Furthermore, molecular formulas were identified, and compound classes were predicted from the features using Sirius version 5.8.3.^35–40^ Details on the instrument method and data processing are described in Supporting Information S2.

##### Female Choice Experiments

Female preference was assessed based on their oviposition preference and their attraction to volatiles emitted by the different OSR accessions. We selected 10 accessions based on the semifield experiment results (Table S1). The five most resistant and the five most susceptible accessions to *D. radicum* larvae were selected. We used four to eight plants from each accession for the experiments, resulting in 60 plants being used for testing. We carried out three independent biological experiments, which we defined below as ‘batches’. The seeds for the batches were sown in 7-day intervals. We used 20 plants per batch and, depending on the germination rate, one to three replicates per accession, ensuring that each OSR accession was represented in each batch. The germination of the plants and the growing conditions are described in Supporting Information S1. For all the following experiments, the plants were used at BBCH stage 15. We conducted two experiments with these plants, the oviposition experiment and the female olfactory preference experiment.

##### Oviposition Experiment

The number of eggs laid on different plant accessions was counted as a proxy of the attractiveness of each OSR accession. The plants of each batch were distributed into five net cages (60 × 40 × 40 cm). Each cage contained four randomly selected plants. Five females and four males were randomly collected from the laboratory insect culture (Supporting Information S1) and placed into each plant cage. The plants were fitted with a felt trap at the base to collect the fly eggs.^41^ After 4 days, we removed the felt traps and counted the number of eggs laid per plant. Because the accessions were tested in randomly chosen groups of four plants in a cage, which may have affected preference and the number of eggs laid on one plant, we used the following scoring system to average out such random effects. The plant with the most eggs in a cage scored three points, the plant with the second most eggs scored two points, and the plant with the third most eggs scored one point. The number of eggs laid was also taken into account. Plants with 1–2 eggs received one additional point, those with 3–5 eggs received two points, with 5–9 eggs three points, with 10–15 eggs four points, and with 16–20 eggs five points. The points from both categories were summed up to give the final score (oviposition score). The two accessions with the highest oviposition score were defined as ‘high oviposition’ on the female preference scale. The four accessions with the lowest score were specified as ‘low oviposition’. Furthermore, we collected the two youngest fully developed leaves. The youngest leaf was used for the wax layer analysis. The second youngest leaf was used for the trichome density analysis.

##### Female Olfactory Preference Experiment

The preference of *D. radicum* females for the volatiles emitted by the different accessions was assessed using a no-choice attractiveness assay. For this no-choice attractiveness assay, we used a linear olfactometer setup similar to Kergunteuil.^24^ We randomly selected four plants for each round. Each plant was covered with a polyamide-polyethylene plastic bag (la.va, Bad Saulgau, Germany) and fixed with a rubber band around the pot. The bag was connected by tubes to the olfactometer, which consisted of a glass tube (length: 60 cm, diameter: 2 cm). The bag also contained a small opening for pressure equalization. A flow meter and a pump were connected after the olfactometer (Figure S1). The pump generated an airflow of 0.4 ± 0.001 l/min (measured after the olfactometer) and pumped the air from the plant through the olfactometer to the point where the female flies were released. The female flies were randomly collected from the laboratory culture, put singly in a 50 mL Falcon tube, and transferred to the greenhouse 1 h before the experiment to acclimatize to the prevailing conditions. The glass tubes were placed on white paper to exclude color effects that may affect the behavior. Two female flies per glass tube were placed in section 1, which was the furthest from the plant (Figures S1). The flies were allowed to move freely in the glass tube. The glass tube was divided into three equal sections (each 20 cm). The female flies were observed for a total of 10 min. The time they spent in each section was monitored. The time spent in section 3 (closest to the plant) was used as a measure of females being attracted to the plant. The time spent in section 1 (close to the point of release) was associated with a lack of response or unattractiveness of the OSR accession. Females without movement within 4 min were excluded from the analysis. The proportion of time that *D. radicum* females spent in section 3 (where they were closest to the volatiles emitted by the *B. napus* accessions) and in section 1 (the fly release section), relative to the 10 min observation period, was calculated and analyzed as described in the statistical analyses section. Each plant was tested with six females in total (3 × 2 females per run). Thus, a total of 24–48 females per accession were tested. To level out diurnal fluctuations, the tests of the different accessions were randomized over the 2 days required to test each batch. As a control, the same setup was used without a plant in the bag. The glass tubes were cleaned with ambient air for 10 min before they were used.

##### Trichomes

Trichomes were examined on four plants per accession from two batches. We counted trichomes within a 1 cm^2^ area on the adaxial surface of the second youngest leaf under a stereomicroscope (Leica DM 1000 LED, 50x). The adaxial surface was selected because females walk and test this side of the leaf. We selected five 1 cm^2^ sections per leaf, three of which included the midrib and two only the lamina. The number of trichomes per cm^2^ was averaged per plant before calculating the average for each accession.

##### Cuticular Wax Layer Metabolome

We weighed the youngest leaf of each plant, measured the leaf length and width and extracted the wax metabolites. Extraction and measurement followed the method of Macel et al.^27^ with some adjustments. In brief, the leaves were carefully dipped into 10 mL chloroform containing 10 μg of tetracosane (Sigma-Aldrich) as the internal standard for 30 s. The leaves were carefully removed and the chloroform evaporated under a stream of nitrogen. After drying completely, the wax residues were resuspended twice in 200 μL chloroform. The entire solution was transferred to a 1.5 mL vial and the chloroform was completely evaporated under a stream of nitrogen. In the next step, the extract was derivatized with 10 μL dried pyridine and 10 μL BSTFA (*N, O*-Bis­(trimethylsilyl)-trifluoroacetamide, Sigma-Aldrich). The vials were sealed with PFTE (phenolic polytetrafluoroethylene) caps and incubated at 80 °C for 1 h. After cooling and evaporation, the samples were resuspended in 100 μL chloroform. Each extraction batch contained an extraction without a leaf as a negative control.

Wax layer compounds were analyzed by GC-MS using a Shimadzu GC-MS-QP2020NX equipped with an autosampler (AOC-20i Plus injector, Shimadzu, China). More details about the method and data processing are described in Supporting Information S3.

##### Volatile Profiling

We passively trapped VOCs on polydimethylsiloxane (PDMS) tubes to test the differences in VOC profiles among the 10 tested OSR accessions. The VOCs were collected from the same plants used for the attractiveness experiment. According to Kallenbach et al. the VOCs were trapped and measured with some modifications.^42^ In brief, plants were individually wrapped in polyester cooking bags (Toppits, Minden, Germany) and fixed with rubber bands around the pot. We placed three cleaned PDMS tubes (diameter: external 1.8 mm, internal 1 mm, length: 10 mm, Carl Roth GmbH, Karlsruhe, Germany) in each bag as technical replicates for each plant. The VOCs were passively absorbed by the PDMS tubes for 24 h. A pot without a plant was trapped simultaneously to exclude the VOC background. In addition, a clean PDMS tube was measured. The VOCs were analyzed by thermal desorption-gas chromatograph-mass spectrometry (TD-GC-MS) using a Shimadzu GCMS-QP2020NX equipped with a thermal desorption unit TD-30R (Shimadzu, Duisburg, Germany). VOCs were separated on a Zebron ZB-5MS column (30 m x 0.25 mm x 0.25 μm, Phenomenex, Torrance, CA, USA). More details are described in Supporting Information S4.

##### Statistical Analyses

All statistical analyses were performed in R version 4.3.0.^43^ The ggplot2 package version 3.4.4 was used to generate all figures unless otherwise noted.^44^ *Larval resistance experiments:* Differences in root damage scores among plant accessions in one experiment were log-transformed and analyzed using a *one-way ANOVA* followed by *Tukey’s HSD test*. Correlations in the root damage among the different experiments (greenhouse vs common garden egg infestation and common garden natural infestation) were analyzed using Spearman’s rank test. Differences in dry root biomass between infested and noninfested plants were analyzed per accession using the *Wilcox test* with a *Bonferroni-*adjusted P-value. The effect of OSR accessions on larval and pupal weight was analyzed using the *Kruskal–Wallis rank sum test* in combination with a *Bonferroni*-adjusted P-value. The root metabolomes of susceptible and resistant accessions were compared by feature-based methods using NMDS with Bray–Curtis distance in the metaMDS function of the vegan package v. 2.6–4 ^45^ followed by *ANOSIM*. Therefore, the four most susceptible and the four most resistant accessions were assigned into separate groups (susceptible and resistant). In addition, a volcano plot was created with the same data using Metaboanalyst v. 6.0 ^46^ and the following settings: fold change >2, *P* < 0.05, including the FDR function, unpaired. The oviposition preference was analyzed using *GLM* with a quasi-Poisson distribution in the R package car. The quality of the model fit was tested using a chi-square test. Larval feeding damage score and oviposition score were compared using *Pearson’s* correlation. The time spent by females in each section of the olfactometer was compared among OSR accessions using *Pearson’s Chi-squared test*. The time spent in different sections within an accession was analyzed using the *Wilcoxon rank sum test* with *Bonferroni*-adjusted P-values. Trichome density was compared among accessions using the *Wilcoxon rank sum test* with *Bonferroni*-adjusted P-values. The peak area of individual wax metabolite features was compared among accessions using the *Kruskal–Wallis test*, followed by *Dunn’s test* as a posthoc test with a *Bonferroni* P-value adjustment. Differences in features between ‘high’ and ‘low’ groups were created as a volcano plot in Metaboanalyst (v. 6.0). The composition of wax metabolites and VOCs among batches, accessions and oviposition categories was analyzed by NMDS with Bray-Curtis distance in the vegan package followed by *ANOSIM*. The peak area of individual VOC features was compared among accessions using *one-way ANOVA* followed by *Tukey’s HSD test*. The homogeneity of variance was tested with *Levene’s test*, the normal distribution of residuals with the *Shapiro test* and visual observation.

## Results

### Larval Resistance in the Roots

#### Semifield Experiment

The manually egg-infested plants and those with natural infestation only, had similar damage levels overall (Spearman’s rank correlation, P-value: 0.012, rho: 0.548, Figure 2). Accessions with the most feeding damage after manual egg infestation were generally receiving more damage due to natural infestation as well (Figure 1, Table S2). Which accessions incurred less damage differed between egg-infested and naturally infested plants.

**Figure 2:**
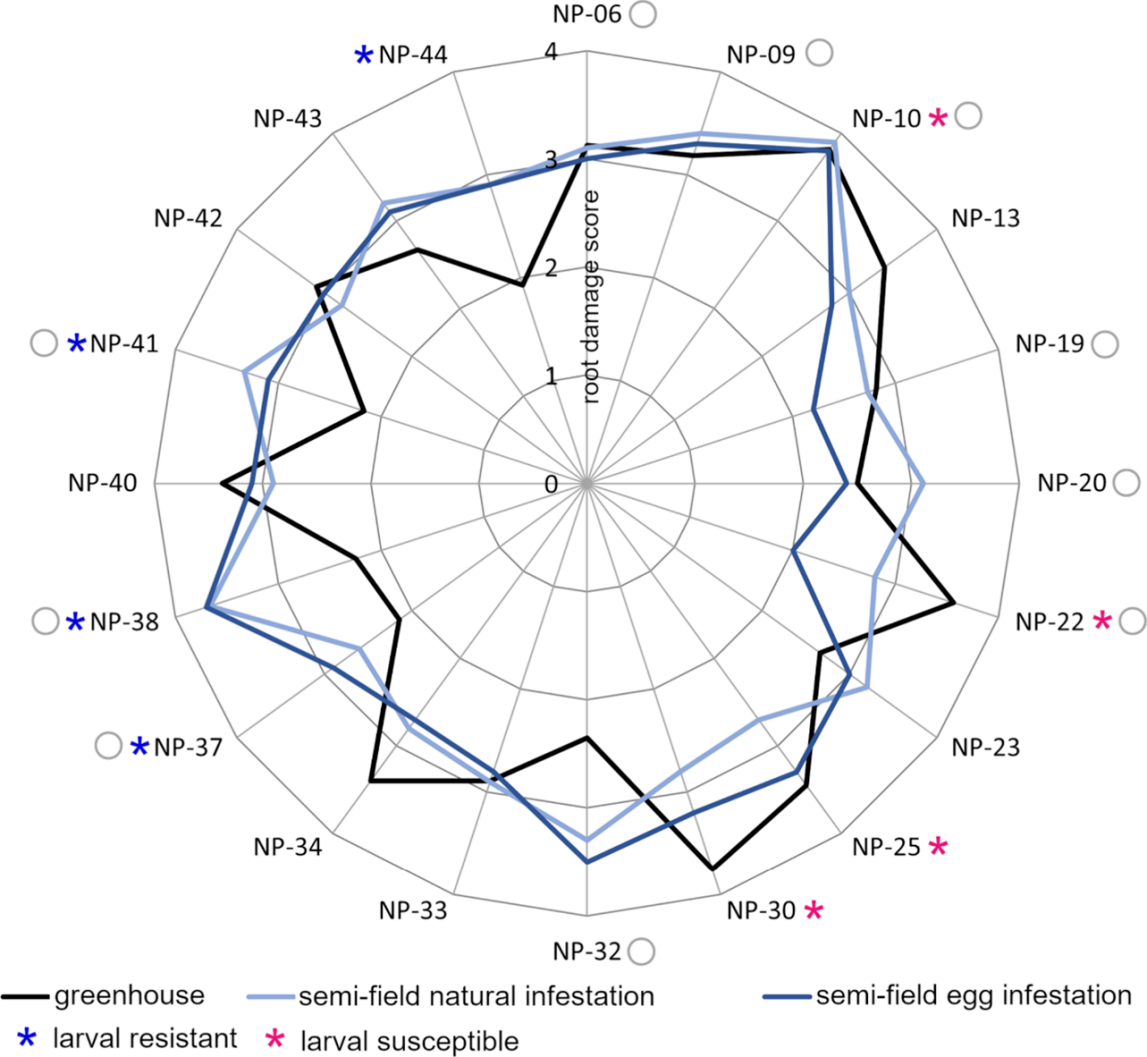
Feeding damage on the roots of 20 *Brassica napus* accessions as assessed in three independent experiments. Damage intensity caused by *Delia radicum* larvae was categorized using a score from 0 – 4 with increasing damage levels. Plants were egg-infested manually (dark blue line) or naturally with *D. radicum* eggs (light blue line) in a semifield experiment or egg-infested manually in a greenhouse experiment (black line). Accessions were defined as larval resistant or susceptible based on the damage level in the greenhouse experiment and labeled with blue or red stars. Accessions selected for the oviposition experiment were labeled with gray circles.

#### Greenhouse Experiment

We repeated the experiment in the greenhouse to assess larval damage under controlled conditions. Here, we found that the accessions NP-10, NP-22, NP-25 and NP-30 (Figure 2, red stars; Tables S3, S4) were the most damaged. Therefore, we refer to them as ‘susceptible accessions’ in the following parts. The least damage was found in accessions NP-37, NP-38, NP-41 and NP-44, which we defined as resistant accessions (Figure 2, blue stars; Tables S3, S4). In general, accessions with low or high feeding damage score in the greenhouse did not receive similar damage scores in the field experiment, except for accession NP-37, which was classified as resistant in the greenhouse and the field trials, and NP-10, which was always severely damaged and thus consistently susceptible (Figure 2).

In line with this, we found no significant correlation between damage occurring in the greenhouse experiment and the egg-infested common garden experiment (Spearman’s rank correlation, P-value: 0.784, rho: 0.065) or between the greenhouse experiment and the natural infestation in the common garden experiment (P-value: 0.456, rho: −0.177).

In addition, we assessed dry root biomass in control and infested plants as a proxy for plant tolerance to damage. We found that dry root biomass was not significantly influenced by *Delia* herbivory in most of the accessions, except for accessions NP-10 (Wilcox test, P = 0.02), NP-25 (P = 0.03) and NP-32 (P = 0.005, Figure S2, Table S3, S4). In these accessions, plant biomass was reduced when *Delia* was feeding on the roots. Two of these accessions, NP-10 and NP-25, belonged to the accessions identified as susceptible.

To determine whether *D. radicum* performance was affected by feeding on different accessions, we counted the number of *D. radicum* larvae and pupae and measured their masses. In general, the number of individuals collected and the ratio of larvae to pupae varied between accessions (Figure 3). Fewer than three larvae were found in the NP-23 and NP-37 accessions, suggesting a low survival rate (Table S3). The highest percentage of surviving individuals was found in NP-44 (43%) and the lowest percentage in NP-23 (1%, Figure 3). Interestingly, none of the larvae feeding on NP-44 roots, the OSR accession with the least root damage, had pupated at the time of collection, while 85% of the collected individuals were in the pupal stage on NP-10, the OSR with the most severe root damage (Figure 3).

**Figure 3:**
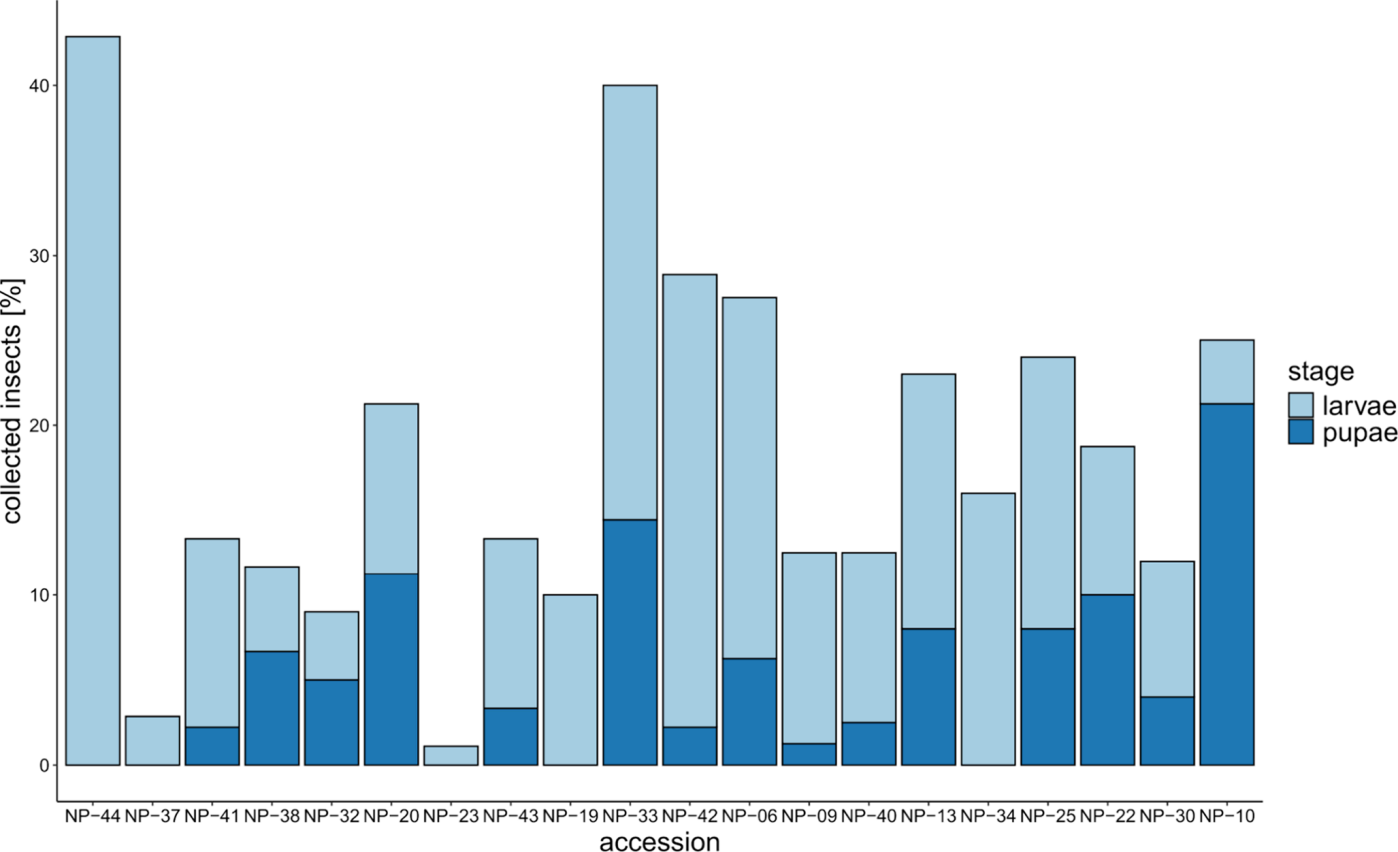
Percentage of recovered *D. radicum* larvae (light blue) and pupae (dark blue) on the roots of 20 different *B. napus* accessions as a proxy for insect performance. Percentages were calculated from the total number of applied eggs (10 per plant x number of replicates (6–10) per accession).

Larval masses were generally similar among the OSR accessions, as were pupal masses (Figure S3, Table S5). Reduced larval weight was found only in the accession with the least root damage level (NP-44, Kruskal–Wallis rank sum test, *P* < 0.02, Figure S3, Table S6).

### Root Metabolic Profile of Selected Resistant and Susceptible Accessions

We analyzed the metabolic profile of noninfested roots from the four accessions, categorized as resistant (NP-37, NP-38, NP-41, and NP-44) as well as the four categorized as susceptible (NP-10, NP-22, NP-25, and NP-30) to *D. radicum* larvae. In total, 833 features were detected over all accessions using LC-qToF-MS in negative mode (Table S7). In an NMDS plot, the metabolomes of the susceptible and resistant accessions separated in two classes (ANOSIM, R = 0.59, P = 0.001) as well as on the level of individual accessions (R = 0.848, P = 0.001, Figure 4a). Especially the metabolic profiles of the resistant accessions NP-41 and NP-44 separated from accessions NP-10, NP-22 and NP-25 (Figure 4a).

**Figure 4:**
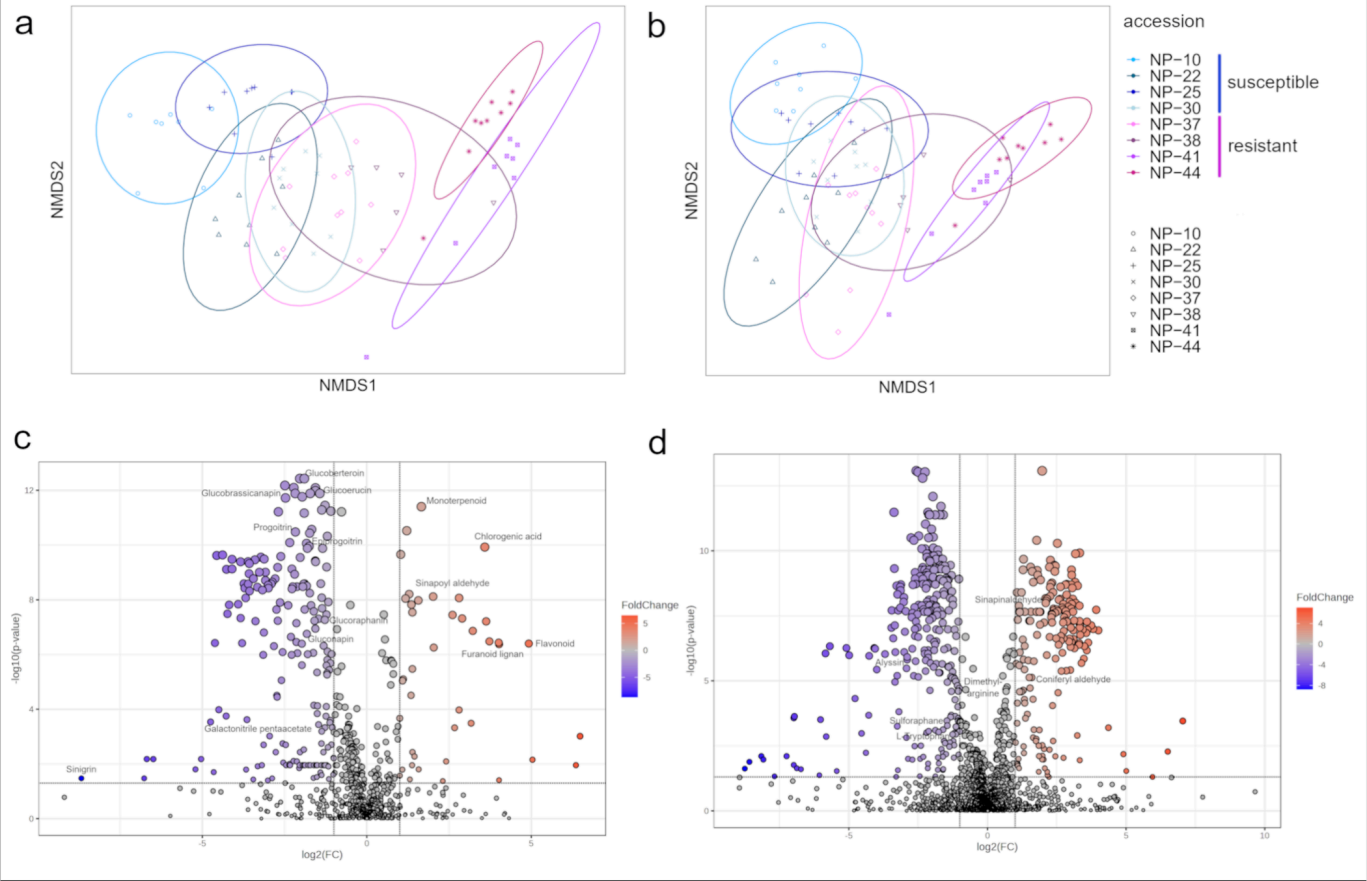
Root metabolome of resistant and susceptible *Brassica napus* accessions without herbivore damage measured in the negative mode (a, c) and positive mode (b, d) of LC-MS. (a, b) The root metabolome is plotted as NMDS of distance based on the peak area with Bray-Curtis distance and colored by accessions (susceptible: red colors, resistant: blue-purple colors). (c, d) Vulcano plot of the log-fold changes in the root metabolome between resistant and susceptible *B. napus* accessions. Each dot represents a feature. Blue dots represent features with significantly lower peak areas (*P* < 0.05, log_2_-fold change < -1.5) in resistant than susceptible accessions. Red dots represent features with a significantly higher peak area (log_2_-fold change > 1.5) in resistant accessions. Labeled features were predicted from an in-house library or Sirius.^35^

When comparing the peak areas of the features, we found 192 features to be reduced in the resistant accessions (Figure 4c, blue dots). These included features predicted as GSLs, several of which we could identify using our in-house reference library. These GSLs reduced in resistant accessions were mainly aliphatic GSLs, such as glucoerucin, glucoraphanin, progoitrin, epiprogoitrin, glucoberteroin, glucobrassicanapin and gluconapin (Figure 4c, log_2_-fold change < −1.4, Table S8). Overall, 41 features were increased in the resistant accessions compared to the susceptible ones (Figure 4c, red dots). These included features predicted as isoflavonoids, lignans, phenylpropanoids, terpenes and metabolites involved in lignin-biosynthesis, such as sinapoyl aldehyde (Figure 4c, log_2_-fold change >2, Table S8). Several of these could also be identified due to a match with our in-house reference library (Table S8).

In the positive mode, we detected a total of 1919 features in the susceptible and resistant accessions together (Table S9). Similar to the negative mode, NMDS analyses also separated the metabolomes acquired in the positive mode (ANOSIM, R = 0.411, P = 0.001, Figure 4b), separating plant accessions on their root metabolome (R = 0.7655, P = 0.001, Figure 4b). Resistant accessions contained 262 features that were lower and 183 that were higher compared to susceptible accessions (Figure 4d). The features that were lower in resistant accessions were mainly predicted as aliphatic GSLs (such as glucoalyssin) or GSL breakdown products (such as sulforaphane), or amino acids (Figure 4d, blue dots, log_2_-fold change < -1.8, Table S8). Features with higher peak areas in resistant accessions were predicted as putative compounds involved in lignin biosynthesis, such as coniferyl-aldehyde (Figure 4d, red dots, log_2_-fold change > 1.5).

### Female Choice and Oviposition

#### Selection of Accessions

For the female choice experiments, we used 10 accessions (Figure 2, gray circles; Table S1), which were selected based on the results of the manually egg-infested semifield experiment. We chose this experiment for its more applied relevance. The three most resistant and susceptible accessions and four randomly selected accessions with intermediate damage were selected for this experiment.

#### Oviposition Experiment

The oviposition preference of *D. radicum* females on 10 different OSR accessions was assessed in a choice experiment. In this experiment, we found significant differences in the oviposition behavior of females in response to different OSR accessions (Figure 5a, S4, Table S10). Female flies preferred the NP-10 and NP-22 accessions for oviposition over the NP-20, NP-32 and NP-37 accessions (Figure 5a, S4, Table S11). Female selection for oviposition tended to correlate positively with larval feeding damage (Figure 5a).

**Figure 5:**
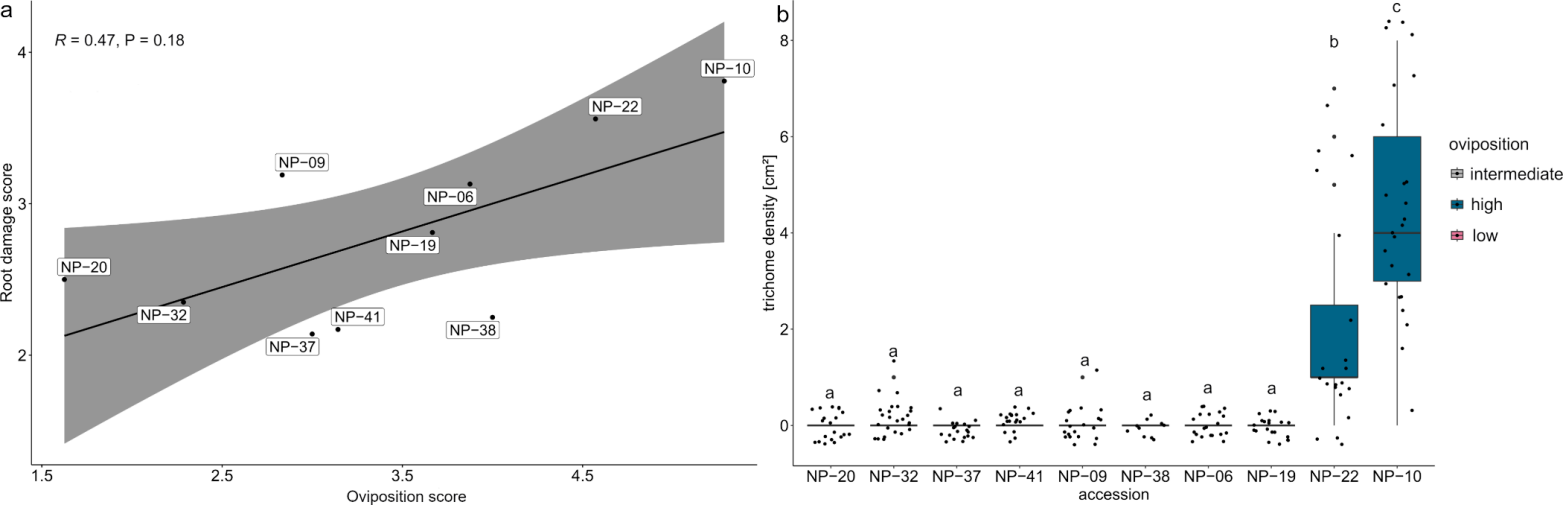
a: Regression of oviposition score of *D. radicum* females on root damage score caused by *D. radicum* larvae in 10 *Brassica napus* accessions. The oviposition score includes the number of eggs laid and the preferred plant among a choice of four plants. b) Trichome density per cm^2^ of leaf in the 10 *B. napus* accessions. Letters indicate significant differences based on the *Wilcoxon rank sum test* with *Bonferroni*-adjusted P-values ≤ 0.05.

#### Female Olfactory Preference Experiment

The behavioral response of females to VOCs was studied using a linear olfactometer setup. We analyzed the attraction of *D. radicum* females to the VOCs of 10 different OSR accessions. In general, between 42 and 69% of the tested females moved in the glass tubes when exposed to VOCs of OSR accessions, except for those exposed to the VOCs of accession NP-06, where only 33% of the females decided to move (Figure 6). Females spent most of their time near the entrance, reflecting a lack of response or unattractiveness of the VOCs (Figure 6, Table S12, S13). The proportion of time spent near the entrance was similar among all accessions (*Pearson’s Chi-squared test*, P = 0.191). This also applied to the time spent in the middle section (P = 0.583, not shown in Figure 6, see Table S12, S13) and the section closest to the plant (P = 0.305). In other words, we did not observe a specific preference or aversion of female flies to specific OSR accessions based on their VOC profile.

**Figure 6:**
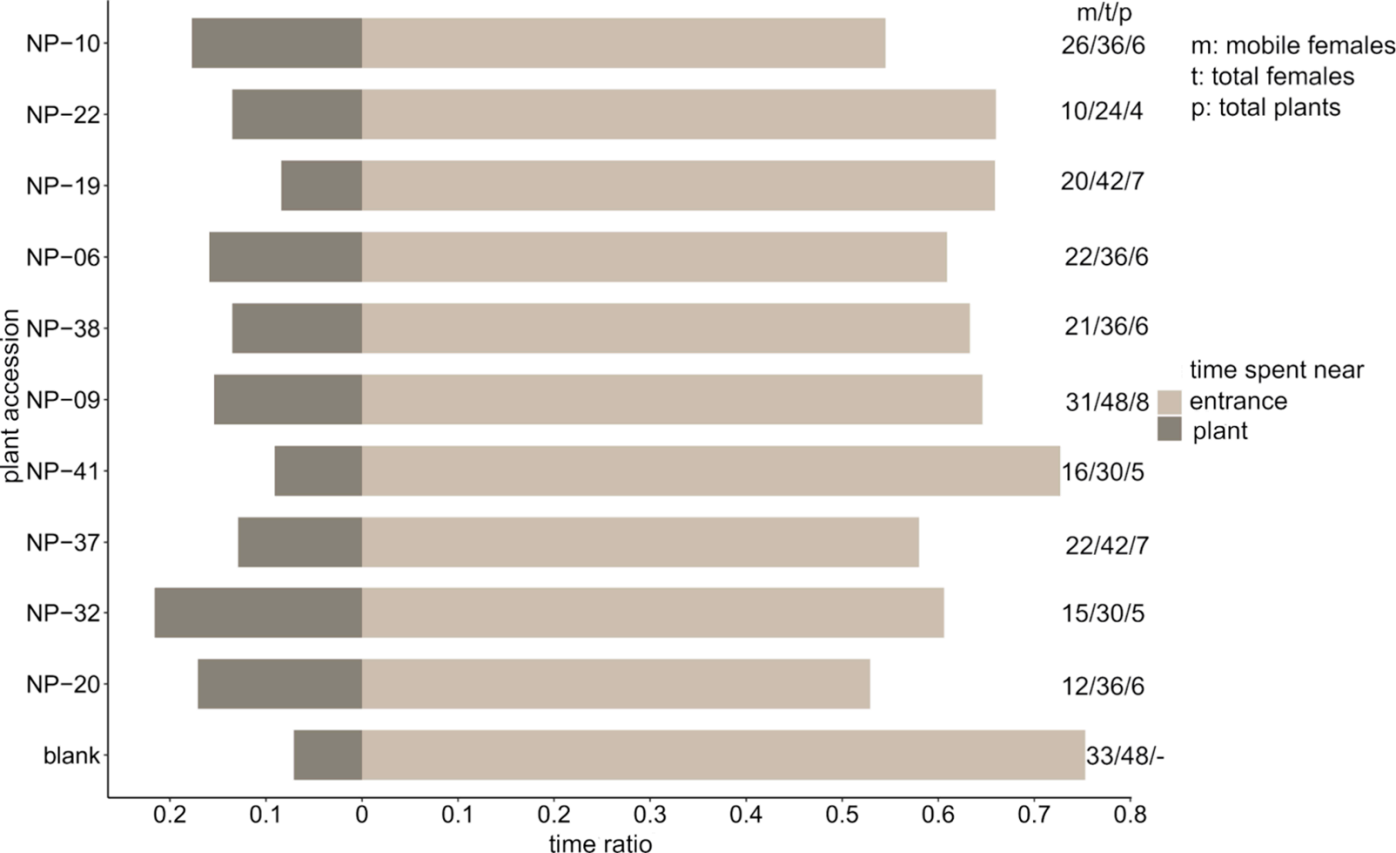
Preference of *Delia radicum* females to volatile organic compounds from 10 different *Brassica napus* accessions in a linear olfactometer setup. The figure presents the proportion of time spent by *D. radicum* females in two sections of a glass tube exposed to *Brassica napus* accessions or pure air. Light brown: Fly entry section; dark brown: Plant airflow entrance. Right side: Mobile females/total tested females/number of plants. Flies were observed for 10 min.

### Mechanisms Underlying Choices

#### Trichomes

Before deciding to oviposit, females explore the leaf texture and quality by walking on the leaf surface. To test whether trichomes on the leaf surface modify oviposition choice, we counted the number of trichomes on the second youngest leaf (Table S14). We found no negative impact of trichome density on oviposition behavior (Figure 5b). Interestingly, the accessions with the highest trichome densities NP-10 and NP-22 (*Wilcoxon rank sum test*, *P* < 0.05, Figure 5b) were the accessions with the highest oviposition scores (Figure 5a).

#### Cuticular Wax Layer Metabolome

To assess whether the chemical composition of the leaf surface may affect the oviposition rate, we examined the metabolome of the cuticular wax layer of the youngest leaf (Table S15). We detected alkanes, long-chain fatty acids, long-chain alcohols, triterpenes, sugars, indole degradation products, nitriles and sulfonamides (Table S16). NMDS analysis did not separate the wax metabolome between accessions with low and high oviposition scores (R = 0.024, P = 0.243) nor among single accessions (*ANOSIM*, R = 0.062, P = 0.061, Figure S5b). A separation occurred among experimental batches (R = 0.243, *P* < 0.001). On a single feature level, we found differences between plant accessions (e.g., feature 46) or features occurring mainly in single accessions (e.g., features 13 and 22, mainly in accession NP-22, Figure S6, Table S17), but no association with oviposition preference.

#### VOC Profiling

Differences in VOC profiles may affect the female oviposition behavior. We measured the VOC composition of the 10 OSR accessions tested (Table S18). We detected mainly monoterpenes, sesquiterpenes, alkanes, alcohols and fatty acids (Table S19). The 10 OSR accessions showed a batch-specific VOC pattern based on the day the VOCs were sampled (*ANOSIM*, R = 0.439, P = 0.001) but no accession-specific clustering (R = 0.001, P = 0.444, Figure S5a). The VOC profiles of accessions with high (NP-22 and NP-10) and low oviposition scores (NP-20 and NP-32, R = 0.01, P = 0.325, Figure S5a) greatly overlapped. While none of the detected features differed significantly between the two oviposition groups, three features (number 10, 41 and 58) differed significantly between the accessions, particularly between NP-09, NP-32, and NP-10 (Figure S7, one-way *ANOVA* followed by *Tukey HSD* test, Table S20).

## Discussion

Ovipositing insect herbivores localize their host plants and assess their quality by combining multiple cues to select the best host for their offspring. In particular, physical and chemical plant signals are involved in these decisions. We studied the feeding and oviposition behavior of *D. radicum* at the intraspecific level of OSR. We found that both feeding damage by *D. radicum* larvae and oviposition choice by females differed among OSR accessions. Roots of lesser-damaged OSR accessions (defined here as resistant accessions) contained fewer GSLs, particularly aliphatic GSLs. At the same time, features involved in lignin biosynthesis were enriched in resistant accessions. The most resistant accessions increased mortality or delayed the development of *D. radicum*. In line with the mother-knows-best hypothesis,^47^ females tended to select those OSR accessions that larvae damaged the most. To study the mechanisms underlying the oviposition preference, we focused on trichomes, wax layer composition and VOCs. We found that the two accessions preferred for oviposition had the highest trichome densities. However, we did not find a relationship between female oviposition preference and cuticular wax layer composition or VOC profile. In general, the VOC and wax metabolome profiles in all OSR accessions greatly overlapped. Together, our results suggest that breeding efforts to increase cabbage root fly resistance may focus on increasing larval resistance, particularly by increasing root lignin and reducing specific root GSLs.

The larvae of *D. radicum* are specialized on the roots of Brassicaceae plants and are constantly exposed to GSLs and their breakdown products.^48^ In order to adapt to the GSL defense system, the larvae detoxify the GSL breakdown products.^15^ In addition to defense, GSLs and their hydrolysis products benefit insect herbivores, in particular, by acting as location cues and feeding stimulants in specialized insects.^7,49–52^ Our data showed that certain GSLs, particularly aliphatic GSLs, promote damage to *D. radicum* larvae. Based on this, we suggest that lowering root concentrations of these GSLs may reduce larval feeding damage of *D. radicum* in OSR roots. Interestingly, cabbage root fly larval performance is negatively affected by the ITCs of the benzenic glucosinolate 2-phenylethylglucosinolate or gluconasturtiin.^15^ The 2-phenylethylglucosinolate is prominently present in *Brassica* roots.^16^ High levels of gluconasturtiin also confer resistance to root-feeding plant pathogenic nematodes^53,54^ and can be selected for breeding programs.^55^ Indole, aliphatic and benzenic GSLs are synthesized in separate biosynthetic pathways governed by different genes and transcription factors.^56^ Thus, it may be possible to select for OSR accessions with high levels of benzenic and low levels of aliphatic GSLs in their roots to optimize resistance to cabbage root fly larvae.

In addition to a lower level of specific GSLs, less damaged OSR roots were enriched with features involved in lignin biosynthesis. Lignin is a complex heterogeneous polymer that improves stability and forms a physical and chemical barrier against herbivores. High lignin content increases root toughness and makes roots more inaccessible and indigestible.^57^ It also increases the penetration time of root-feeding insects.^58^ We expected that OSR accessions with low lignin-related features would be more damaged by larvae and reduce root biomass due to faster penetration time and better/faster digestion of root material. Our assumptions were confirmed for the most resistant and susceptible accession. While the most susceptible accession showed lower root biomass and improved insect development in time, we observed lower larval development, weight and survival in the two most resistant accessions. Our results thus suggest that breeding for high lignin content, or woody roots, may be a viable option to obtain high natural resistance to OSR. However, whether this does not result in trade-offs with root growth or yield should be considered. In addition, the stubble of woody OSR plants may cause issues after harvesting.

Another way to increase resistance is to affect the oviposition choice of female flies. Specialized insects such as *D. radicum* filter out their host plants in the background of various nonhost plants by recognizing specific plant cues. Apart from visual cues such as leaf shape and color,^21,59^ the oviposition behavior of *D. radicum* females is influenced by the physical structures and chemical compositions of the leaf surface.^21,22,60^ After landing on the leaves, the females probe the leaf by walking on the surface.^61^

Mechanical structures impeding herbivorous insects, such as trichomes, are present on the leaf surface. They form a physical and in the case of glandular trichomes chemical barrier against herbivorous insects. In addition to their feeding deterrent function, trichomes can deter insects from oviposition.^62–64^ For instance, *Plutella xylostella*, a moth specialized in Brassicaceae, preferred *Arabidopsis thaliana* leaves with low trichome densities for egg deposition.^65^ Here we found that trichomes do not deter cabbage root fly females. In fact, accessions with higher trichome densities received the most eggs. Nonglandular trichomes of *A. thaliana*, a relative of OSR, contain aliphatic and indole GSLs.^66^ Possibly, the trichomes of OSR also contain these GSLs. Due to the stimulatory effect of GSLs and their breakdown products on the oviposition behavior of *D. radicum*, GSL-containing trichomes may explain the preference for oviposition in our experiment.^67^ In addition, low trichome densities on the leaf may indicate a higher chance of infestation by aboveground insects. It was shown that aboveground herbivore damage on *Brassica* species negatively affects belowground herbivores and their natural enemies via systemically induced responses.^68^ Induction by aboveground herbivores also changes GSL levels in the roots, which may impact the performance of *D. radicum* larvae.^69^

During the female’s leaf walk, the insects probe the chemical composition of the leaf surface in addition to mechanical structures. Compounds on the cuticular wax layer stimulate sensory neurons on the tarsal sensilla that contribute to oviposition decisions.^22^ The plant wax layer generally protects against biotic and environmental stresses including herbivore damage, pathogen infection and UV radiation.^70–72^ According to Zhang et al. it is mainly composed of long-chain fatty acids, alkanes, alcohols, ketones, and terpenoids, which is in line with the results of our studies.^73^ In addition, the wax layer can trigger insect feeding and oviposition behavior.^72,74^ Removal or disruption of lipid structures resulted in increased oviposition by the butterfly *Plutella xylostella* on *B. oleracea* plants.^75^

Apart from GSLs, phytochemicals on the leaf surface impact the preference of *D. radicum* females. For instance, thia-triaza-fluorenes (TTF, 1,2-dihydro-3-thia-4,10,10b-triazacyclo-penta­[.a.]­fluorine-1-carboxylic acid) stimulate the oviposition in *D. radicum*.^60,76^ Here, we examined the chemical composition of the cuticular wax layer on different OSR accessions. However, we found no correlation between egg deposition and cuticular wax layer metabolites. Overall, the wax layer metabolome was similar among OSR accessions with high and low oviposition scores. In addition, we did not detect the oviposition stimulator TTF in the OSR accessions used, which may be due to the very low concentrations of this compound.^60^ In order to identify metabolites that influence the oviposition selection of the cabbage root fly females in OSR at lower concentrations, more leaf biomass would have to be harvested.

Other phytochemicals affecting the preference of *D. radicum* females are phytoalexins, which are structurally related to indole GSLs, especially methoxybrassinin, cyclobrassinin and brassitin, which are mainly known for their antimicrobial function, also stimulate oviposition behavior in *D. radicum*.^77^ As we did not identify these compounds in our LC-MS analyses, they may not be present in high enough concentrations to impact oviposition in the selected OSR accessions.

Plant-emitted odors are other relevant cues that govern the oviposition behavior of *D. radicum* females. In preference assays using different Brassicaceae, it was found that they are attracted to allyl isothiocyanate, (*Z*)-3-hexenyl acetate, 1,8-cineole, linalool, β-caryophyllene, α-humulene, α-farnesene.^24,78^ Other VOCs negatively affect attraction and oviposition behavior, including dimethyl disulfides at high concentrations and salicylaldehydes.^78,79^

Based on our VOC analysis and choice assays with cabbage root fly females, we did not find a strong preference for a particular OSR accession. The observed lack of female preference may be due to the similar VOC profiles among the OSR accessions used. Most of the attractive and repellent oviposition compounds mentioned in the literature (see above) were not detected or were similar in peak area among the OSR accessions. The absence of these VOCs in our analysis may be due to the passive trapping method, which is less sensitive than dynamic headspace sampling used in other studies.^24,80^ Possibly, cabbage root fly females use VOCs for global host-plant recognition, e.g. to detect that the plant is an OSR individual, rather than as a cue to identify the most suitable host plant for oviposition.^24,81^ In line with this assumption, we observed batch effects for both VOCs and wax layer metabolites. Both traits appear highly dependent on the environment^82^ or other random factors. Therefore, they may not be reliable parameters for finding the most suitable host plants for cabbage root fly oviposition. Further studies should consider that besides leaf traits, soil properties such as texture (loose structures), moisture and light intensity influence the oviposition selection.^23,83,84^ In addition, volatiles released by the roots and possibly by soil microorganisms alter oviposition choice.^23,85^ Together, these different factors may determine the final decision made by *D. radicum* females.

Understanding the resistance of OSR to *D. radicum* is of fundamental interest for plant breeding and crop protection. Our results suggest that breeders should focus on breeding OSR with woody roots and low aliphatic GSL to increase the OSR resistance to *D. radicum*. However, our findings have to be confirmed under field conditions. In addition, it should be considered that breeding for resistance to *D. radicum* may trade off with resistance to other herbivores as well as with agronomic traits, such as seed production and oil quality. By screening more accessions for resistance against *D. radicum* in OSR as well as coinfestation with additional above- and belowground pests (e.g., nematodes, endomopathogenic fungi, caterpillars and beetles), we may identify such potential trait-offs. We also found that multiple (chemical) traits in OSR accessions may contribute to *D. radicum* preference and performance. Probably, it is the combination of these OSR traits conferring *D. radicum* resistance in agrosystems. This knowledge will help us to develop sustainable and environmentally friendly strategies to protect OSR in the field. In addition, the exploration of resistance mechanisms in natural *Brassica* species has the potential to facilitate the identification of additional resistance traits and incorporate these into breeding approaches.^86^ Additional research is needed into the factors determining oviposition choice among OSR accessions by *D. radicum* females. In this case, studies on leaf toughness, stem structure, root volatile emission and soil microbiome communities are relevant. Thus, it is important to focus on both agronomically relevant life stages in the development of novel approaches for environmentally friendly control of *D. radicum* in OSR.

## Supplementary Material





## Data Availability

Raw data from the wax layer metabolome (GC-MS) are available at DOI 10.5281/zenodo.15010174, from the volatile organic compound (GC-MS) at DOI 10.5281/zenodo.15007332, and raw and processed data from the root metabolome (LC-MS) are available at DOI 10.5281/zenodo.15010257.

## Notes

Additional experimental details, materials, methods, data processing, and figures of experimental design, plant and insect performance, metabolites and volatiles (PDF)
Additional tables of the accessions, root damage, insect performance and preference, plant metabolites, trichomes and volatile organic compounds (XLSX)

## Abbreviations

GC-MS: Gas chromatography–mass spectrometry
GSL: Glucosinolate
iDiv: German Centre for Integrative Biodiversity research
LC-MS: Liquid chromatography–mass spectrometry
OSR: Oilseed rape
PDA: Photodiode array detector
PDMS: Polydimethylsiloxane
UHPLC: Ultrahigh-performance liquid chromatography
VOC: Volatile organic compounds

## References

[1] Zheng X., Koopmann B., Ulber B., von Tiedemann A. (2020). A Global Survey
on Diseases and Pests in Oilseed Rape—Current Challenges and
Innovative Strategies of Control. Front. Agron..

[2] Sponsler D. B., Grozinger C. M., Hitaj C., Rundlöf M., Botías C., Code A., Lonsdorf E. V., Melathopoulos A. P., Smith D. J., Suryanarayanan S. (2019). Pesticides and pollinators:
A socioecological synthesis. Science of The
Total Environment.

[3] Kalia A., Gosal S. K. (2011). Effect of pesticide
application on soil microorganisms. Archives
of Agronomy and Soil Science.

[4] Hao Z.-P., Zhan H.-X., Gao L.-L., Huang F., Zhu L.-N., Hou S.-M. (2020). Possible effects
of leaf tissue characteristics of
oilseed rape Brassica napus on probing and feeding behaviors of cabbage
aphids Brevicoryne brassicae. Arthropod-Plant
Interactions.

[5] Schaefer H. L., Brandes H., Ulber B., Becker H. C., Vidal S. (2017). Evaluation
of nine genotypes of oilseed rape (Brassica napus L.) for larval infestation
and performance of rape stem weevil (Ceutorhynchus napi Gyll.). PLoS One.

[6] Eickermann M., Ulber B., Vidal S. (2011). Resynthesized
lines and cultivars
of Brassica napus L. provide sources of resistance to the cabbage
stem weevil (Ceutorhynchus pallidactylus (Mrsh.)). Bulletin of Entomological Research.

[7] Hopkins R. J., van Dam N. M., van Loon J. J. A. (2009). Role
of Glucosinolates in Insect-Plant
Relationships and Multitrophic Interactions. Annu. Rev. Entomol..

[8] Burow M., Losansky A., Müller R., Plock A., Kliebenstein D. J., Wittstock U. (2008). The Genetic
Basis of Constitutive and Herbivore-Induced
ESP-Independent Nitrile Formation in Arabidopsis. Plant Physiol..

[9] Jeschke, V. ; Gershenzon, J. ; Vassão, D. G. Metabolism of Glucosinolates and Their Hydrolysis Products in Insect Herbivores. In The Formation, Structure and Activity of Phytochemicals, Jetter, R. , Ed.; Springer International Publishing: 2015; pp 163–194.

[10] Ratzka A., Vogel H., Kliebenstein D. J., Mitchell-Olds T., Kroymann J. (2002). Disarming the mustard oil bomb. Proc. Natl. Acad. Sci. U. S. A..

[11] Huang X., Renwick J. A. A. (1994). Relative activities
of glucosinolates as oviposition
stimulants forPieris rapae andP. napi oleracea. Journal of Chemical Ecology.

[12] Yang J., Guo H., Jiang N.-J., Tang R., Li G.-C., Huang L.-Q., van Loon J. J. A., Wang C.-Z. (2021). Identification of a gustatory receptor
tuned to sinigrin in the cabbage butterfly Pieris rapae. PLOS Genetics.

[13] Dosdall L. M., Good A., Keddie B. A., Ekuere U., Stringam G. (2000). Identification
and evaluation of root maggot (Delia spp.) (Diptera: Anthomyiidae)
resistance within Brassicaceae. Crop Prot..

[14] Niemann J., Szwarc J., Bocianowski J., Weigt D., Mrówczyński M. (2020). In-field screening
for host plant resistance to Delia radicum and Brevicoryne brassicae
within selected rapeseed cultivars and new interspecific hybrids. Open Life Sci..

[15] Sontowski R., Guyomar C., Poeschl Y., Weinhold A., van Dam N. M., Vassão D. G. (2022). Mechanisms of Isothiocyanate Detoxification in Larvae
of Two Belowground Herbivores, Delia radicum and D. floralis (Diptera:
Anthomyiidae). Front. Physiol..

[16] Tsunoda T., Krosse S., van Dam N. M. (2017). Root and
shoot glucosinolate allocation
patterns follow optimal defence allocation theory. Journal of Ecology.

[17] Crespo E., Hordijk C. A., de Graaf R. M., Samudrala D., Cristescu S. M., Harren F. J. M., van
Dam N. M. (2012). On-line detection
of root-induced volatiles in Brassica nigra plants infested with Delia
radicum L. root fly larvae. Phytochemistry.

[18] Dugger C. D., Lightle D., Matteson M., Rasmussen A., Buckland K., Isman M. (2024). Efficacy of conventional
and organic
pesticides following ingestion by Delia radicum (Diptera: Anthomyiidae). J. Econ. Entomol..

[19] Neveu N., Grandgirard J., Nenon J. P., Cortesero A. M. (2002). Systemic
Release of Herbivore-Induced Plant Volatiles by Turnips Infested by
Concealed Root-Feeding Larvae Delia radicum L. Journal of Chemical Ecology.

[20] Kostal V., Finch S. (1994). Influence of background
on host-plant selection and subsequent oviposition
by the cabbage root fly (Delia radicum). Entomologia
Experimentalis et Applicata.

[21] Roessingh P., Städler E. (1990). Foliar form,
colour and surface characteristics influence
oviposition behaviour in the cabbage root fly Delia radicum. Entomologia Experimentalis et Applicata.

[22] Roessingh P., Städler E., Fenwick G. R., Lewis J. A., Nielsen J. K., Hurter J., Ramp T. (1992). Oviposition and tarsal chemoreceptors
of the cabbage root fly are stimulated by glucosinolates and host
plant extracts. Entomologia Experimentalis et
Applicata.

[23] Kostal V., Baur R., Stadler E. (2000). Exploration
and assessment of the
oviposition substrate by the cabbage root fly, Delia radicum (Diptera:
Anthomyiidae). European Journal of Entomology.

[24] Kergunteuil A., Dugravot S., Danner H., van Dam N. M., Cortesero A. M. (2015). Characterizing
Volatiles and Attractiveness of Five Brassicaceous Plants with Potential
for a ‘Push-Pull’ Strategy Toward the Cabbage Root Fly,
Delia radicum. Journal of Chemical Ecology.

[25] van
Dam N. M., Samudrala D., Harren F. J. M., Cristescu S. M. (2012). Real-time
analysis of sulfur-containing volatiles in Brassica plants infested
with root-feeding Delia radicum larvae using proton-transfer reaction
mass spectrometry. AoB Plants.

[26] Tuttle A. F., Ferro D. N., Idoine K. (1988). Role of visual and
olfactory stimuli
in host finding of adult cabbage root flies. Delia radicum. Entomologia Experimentalis et Applicata.

[27] Macel M., Visschers I. G. S., Peters J. L., van Dam N. M., de Graaf R. M. (2020). High Concentrations
of Very Long Chain Leaf Wax Alkanes of Thrips Susceptible Pepper Accessions
(Capsicum spp). Journal of Chemical Ecology.

[28] Müller C., Hilker M. (2001). Host Finding and Oviposition Behavior in a Chrysomelid
Specialist--the Importance of Host Plant Surface Waxes. Journal of Chemical Ecology.

[29] Grosser K., van Dam N. M. (2017). A Straightforward
Method for Glucosinolate Extraction
and Analysis with High-pressure Liquid Chromatography (HPLC). J. Visualized Exp..

[30] Lancashire P. D., Bleiholder H., Boom T. V. D., Langelüddeke P., Stauss R., Weber E., Witzenberger A. (1991). A uniform
decimal code for growth stages of crops and weeds. Ann. Appl. Biol..

[31] Danner H., Brown P., Cator E. A., Harren F. J. M., van
Dam N. M., Cristescu S. M. (2015). Aboveground and Belowground Herbivores
Synergistically Induce Volatile Organic Sulfur Compound Emissions
from Shoots but Not from Roots. Journal of Chemical
Ecology.

[32] Weinhold A., Döll S., Liu M., Schedl A., Pöschl Y., Xu X., Neumann S., van Dam N. M. (2022). Tree species
richness differentially
affects the chemical composition of leaves, roots and root exudates
in four subtropical tree species. Journal of
Ecology.

[33] Schmid R., Heuckeroth S., Korf A., Smirnov A., Myers O., Dyrlund T. S., Bushuiev R., Murray K. J., Hoffmann N., Lu M. (2023). Integrative analysis of multimodal mass spectrometry
data in MZmine 3. Nat. Biotechnol..

[34] Wang M., Carver J. J., Phelan V. V., Sanchez L. M., Garg N., Peng Y., Nguyen D. D., Watrous J., Kapono C. A., Luzzatto-Knaan T. (2016). Sharing and community curation of mass spectrometry
data with Global Natural Products Social Molecular Networking. Nat. Biotechnol..

[35] Dührkop K., Fleischauer M., Ludwig M., Aksenov A. A., Melnik A. V., Meusel M., Dorrestein P. C., Rousu J., Böcker S. (2019). SIRIUS 4:
a rapid tool for turning tandem mass spectra into metabolite structure
information. Nat. Methods.

[36] Dührkop K., Nothias L.-F., Fleischauer M., Reher R., Ludwig M., Hoffmann M. A., Petras D., Gerwick W. H., Rousu J., Dorrestein P. C. (2021). Systematic classification of unknown metabolites
using high-resolution fragmentation mass spectra. Nat. Biotechnol..

[37] Kim H. W., Wang M., Leber C. A., Nothias L.-F., Reher R., Kang K. B., van der Hooft J. J. J., Dorrestein P. C., Gerwick W. H., Cottrell G. W. (2021). NPClassifier:
A Deep Neural Network-Based
Structural Classification Tool for Natural Products. J. Nat. Prod..

[38] Djoumbou
Feunang Y., Eisner R., Knox C., Chepelev L., Hastings J., Owen G., Fahy E., Steinbeck C., Subramanian S., Bolton E. (2016). ClassyFire: automated
chemical classification with a comprehensive, computable taxonomy. Journal of Cheminformatics.

[39] Dührkop K., Shen H., Meusel M., Rousu J., Böcker S. (2015). Searching
molecular structure databases with tandem mass spectra using CSI:FingerID. Proc. Natl. Acad. Sci. U. S. A..

[40] Ludwig M., Nothias L.-F., Dührkop K., Koester I., Fleischauer M., Hoffmann M. A., Petras D., Vargas F., Morsy M., Aluwihare L. (2019). ZODIAC: database-independent molecular formula
annotation using Gibbs sampling reveals unknown small molecules. bioRxiv.

[41] Kergunteuil A., Dugravot S., Mortreuil A., Le Ralec A., Cortesero A. M. (2012). Selecting
volatiles to protect brassicaceous crops against the cabbage root
fly, Delia radicum. Entomologia Experimentalis
et Applicata.

[42] Kallenbach M., Oh Y., Eilers E. J., Veit D., Baldwin I. T., Schuman M. C. (2014). A robust,
simple, high-throughput technique for time-resolved plant volatile
analysis in field experiments. Plant Journal.

[43] R Core Team A language and environment for statistical computing. 4.3.0 ed.; R Foundation for Statistical Computing: Vienna, Austria, 2023.

[44] Wickham, H. Getting Started with ggplot2. In ggplot2: Elegant Graphics for Data Analysis; Springer International Publishing: 2016; pp 11–31.

[45] vegan: Community Ecology Package; R package: 2022. https://CRAN.R-project.org/package=vegan (accessed.

[46] Pang Z., Lu Y., Zhou G., Hui F., Xu L., Viau C., Spigelman A. F., MacDonald P. E., Wishart D. S., Li S., Xia J. (2024). MetaboAnalyst 6.0: towards a unified platform for metabolomics
data processing, analysis and interpretation. Nucleic Acids Res..

[47] Valladares G., Lawton J. H. (1991). Host-Plant Selection in the Holly Leaf-Miner: Does
Mother Know Best?. J. Anim. Ecol..

[48] Finch S., Ackley C. M. (1977). Cultivated and wild host plants supporting
populations
of the cabbage root fly. Annals of Applied Biology.

[49] Rodman J. E., Chew F. S. (1980). Phytochemical correlates of herbivory in a community
of native and naturalized cruciferae. Biochemical
Systematics and Ecology.

[50] Bartlet E., Parsons D., Williams I. H., Clark S. J. (1994). The influence of
glucosinolates and sugars on feeding by the cabbage stem flea beetle,
Psylliodes chrysocephala. Entomologia Experimentalis
et Applicata.

[51] Arany A. M., de Jong T. J., Kim H. K., van Dam N. M., Choi Y. H., Verpoorte R., van der Meijden E. (2008). Glucosinolates and other metabolites
in the leaves of Arabidopsis thaliana from natural populations and
their effects on a generalist and a specialist herbivore. Chemoecology.

[52] Jeschke V., Kearney E. E., Schramm K., Kunert G., Shekhov A., Gershenzon J., Vassão D. G. (2017). How Glucosinolates
Affect Generalist
Lepidopteran Larvae: Growth, Development and Glucosinolate Metabolism. Front. Plant Sci..

[53] Potter M. J., Vanstone V. A., Davies K. A., Kirkegaard J. A., Rathjen A. J. (1999). Reduced Susceptibility of Brassica napus to Pratylenchus
neglectus in Plants with Elevated Root Levels of 2-Phenylethyl Glucosinolate. J. Nematol..

[54] Kabouw P., van der Putten W. H., van Dam N. M., Biere A. (2010). Effects of intraspecific
variation in white cabbage (Brassica oleracea var. capitata) on soil
organisms. Plant and Soil.

[55] Potter M. J., Vanstone V. A., Davies K. A., Rathjen A. J. (2000). Breeding to Increase
the Concentration of 2-phenylethyl Glucosinolate in the Roots of Brassica
napus. Journal of Chemical Ecology.

[56] Mitreiter S., Gigolashvili T. (2020). Regulation of glucosinolate biosynthesis. J. Exp. Bot..

[57] Moore B. D., Johnson S. N. (2017). Get Tough, Get Toxic, or Get a Bodyguard: Identifying
Candidate Traits Conferring Belowground Resistance to Herbivores in
Grasses. Front. Plant Sci..

[58] Johnson S. N., Hallett P. D., Gillespie T. L., Halpin C. (2010). Below-ground herbivory
and root toughness: a potential model system using lignin-modified
tobacco. Physiological Entomology.

[59] Prokopy R. J., Collier R. H., Finch S. (1983). Leaf Color
Used by Cabbage Root Flies
to Distinguish Among Host Plants. Science.

[60] Hurter J., Ramp T., Patrian B., Städler E., Roessingh P., Baur R., de Jong R., Nielsen J. K., Winkler T., Richter W. J. (1999). Oviposition stimulants
for the cabbage root fly: isolation from cabbage leaves. Phytochemistry.

[61] Schoonhoven, L. ; Jermy, T. ; Van Loon, J. Host-plant selection: how to find a host plant. In Insect-plant biology, Vol. 2nd ed.; Oxford University Press: 2005; pp 121–153.

[62] Plett J. M., Wilkins O., Campbell M. M., Ralph S. G., Regan S. (2010). Endogenous
overexpression of Populus MYB186 increases trichome density, improves
insect pest resistance, and impacts plant growth. Plant Journal.

[63] Tian D., Tooker J., Peiffer M., Chung S. H., Felton G. W. (2012). Role of
trichomes in defense against herbivores: comparison of herbivore response
to woolly and hairless trichome mutants in tomato (Solanum lycopersicum). Planta.

[64] Shanower, T. G. Trichomes and Insects. In Encyclopedia of Entomology; Springer Netherlands: 2005; pp 2333–2335.

[65] Handley R., Ekbom B., Ågren J. (2005). Variation in trichome density and
resistance against a specialist insect herbivore in natural populations
of Arabidopsis thaliana. Ecological Entomology.

[66] Frerigmann H., Böttcher C., Baatout D., Gigolashvili T. (2012). Glucosinolates
are produced in trichomes of Arabidopsis thaliana. Front Plant Sci..

[67] Roessingh P., Städler E., Baur R., Hurter J., Ramp T. (1997). Tarsal chemoreceptors
and oviposition behaviour of the cabbage root fly (Delia radicum)
sensitive to fractions and new compounds of host-leaf surface extracts. Physiological Entomology.

[68] Soler R., Bezemer T. M., Cortesero A. M., Van der Putten W. H., Vet L. E. M., Harvey J. A. (2007). Impact of foliar
herbivory on the
development of a root-feeding insect and its parasitoid. Oecologia.

[69] van
Dam N. M., Witjes L., Svatoš A. (2004). Interactions
between aboveground and belowground induction of glucosinolates in
two wild Brassica species. New Phytologist.

[70] Long L. M., Patel H. P., Cory W. C., Stapleton A. E. (2003). The maize
epicuticular wax layer provides UV protection. Functional Plant Biology.

[71] Favaro M. A., Molina M. C., Roeschlin R. A., Gadea J., Gariglio N., Marano M. R. (2020). Different Responses
in Mandarin Cultivars Uncover a
Role of Cuticular Waxes in the Resistance to Citrus Canker. Phytopathology®.

[72] Rebora M., Salerno G., Piersanti S., Gorb E., Gorb S. (2020). Role of Fruit
Epicuticular Waxes in Preventing Bactrocera oleae (Diptera: Tephritidae)
Attachment in Different Cultivars of Olea europaea. Insects.

[73] Zhang Y.-L., You C.-X., Li Y.-Y., Hao Y.-J. (2020). Advances in Biosynthesis,
Regulation, and Function of Apple Cuticular Wax. Front. Plant Sci..

[74] Eigenbrode S. D., Espelie K. E. (1995). Effects of Plant
Epicuticular Lipids on Insect Herbivores. Annu.
Rev. Entomol..

[75] Uematsu H., Sakanoshita A. (1989). Possible Role
of Cabbage Leaf Wax Bloom in Suppressing
Diamondback Moth Plutella xylostella­(Lepidoptera: Yponomeutidae) Oviposition. Appl. Entomol. Zool..

[76] Städler E., Reifenrath K. (2009). Glucosinolates on the leaf surface perceived by insect
herbivores: review of ambiguous results and new investigations. Phytochemistry Reviews.

[77] Baur R., Städler E., Monde K., Takasugi M. (1998). Phytoalexins from Brassica
(Cruciferae) as oviposition stimulants for the cabbage root fly, Delia
radicum. CHEMOECOLOGY.

[78] Lamy F., Dugravot S., Cortesero A. M., Chaminade V., Faloya V., Poinsot D. (2018). One more step toward
a push-pull
strategy combining both a trap crop and plant volatile organic compounds
against the cabbage root fly Delia radicum. Environmental Science and Pollution Research.

[79] den
Ouden H., Alkema D. P. W., Klijnstra J. W., Theunissen J., de Vlieger J. J. (1997). Preference and non-preference experiments
with aerial repellents against Delia radicum L (Dipt., Anthomyiidae)
in a wind tunnel. Journal of Applied Entomology.

[80] Soler R., Harvey J. A., Kamp A. F. D., Vet L. E. M., Van
der Putten W. H., Van Dam N. M., Stuefer J. F., Gols R., Hordijk C. A., Martijn Bezemer T. (2007). Root herbivores influence the behaviour
of an aboveground parasitoid through changes in plant-volatile signals. Oikos.

[81] Nottingham S. F. (1988). Host-plant
finding for oviposition by adult cabbage root fly, Delia radicum. Journal of Insect Physiology.

[82] Holopainen J. K., Gershenzon J. (2010). Multiple stress
factors and the emission of plant VOCs. Trends
Plant Sci..

[83] Traynier R. M. M. (1967). Stimulation
of oviposition by the cabbage root fly Erioischia brassicae. Entomologia Experimentalis et Applicata.

[84] Zohren E. (1968). Laboruntersuchungen
zu Massenanzucht, Lebensweise, Eiablage und Eiablageverhalten der
Kohlfliege, Chortophila brassicae Bouché (Diptera, Anthomyiidae). Zeitschrift für Angewandte Entomologie.

[85] Ellis, P. R. ; Taylor, J. D. ; Littlejohn, I. H. The role of microorganisms colonising radish seedlings in the oviposition behaviour of cabbage root fly, Delia radicum. In Proceedings of the 5th International Symposium on Insect Plant Relationships, Wageningen, the Netherlands, 1982, pp 131–137.

[86] Shuhang W., Voorrips R. E., Steenhuis-Broers G., Vosman B., van Loon J. J. A. (2016). Antibiosis
resistance against larval cabbage root fly, Delia radicum, in wild
Brassica-species. Euphytica.

