# Supplementary material for: Natural Resistance to and Attraction of the Cabbage Root Fly () in Oilseed Rape Accessions
Source: J Agric Food Chem. 2025 Jul 2;73(28):17494–506. doi: 10.1021/acs.jafc.5c03948 (PMC12272681; doi:10.1021/acs.jafc.5c03948)
Supplement: Supplementary file 1 [file jf5c03948_si_001.pdf]

Supporting information

# NATURAL RESISTANCE TO AND ATTRACTION OF THE CABBAGE ROOT FLY (*Delia radicum*) IN OILSEED RAPE ACCESSIONS

Rebekka Sontowski[1,2,3*], Amine Abbadi[4], Hannah Koller[1,3], Steffen Rietz[4], Andreas Schedl[1,3,5], Alexander Weinhold[1,3], Nicole M. van Dam[1,2,3]

[1] Friedrich Schiller University Jena, Dornburger Str. 143, Jena, Germany

[2] Leibnitz Institute of Vegetable and Ornamental Crops (IGZ) , Theodor-Echtermeyer-Weg 1, 14979 Großbeeren, Germany

[3] German Centre for Integrative Biodiversity Research (iDiv) Halle-Jena-Leipzig, Puschstr. 4, 04103 Leipzig, Germany

[4] NPZ Innovation GmbH, Hohenlieth-Hof 1, 24363 Holtsee, Germany

[5] DBFZ Deutsches Biomasseforschungszentrum gemeinnützige GmbH, Torgauer Straße 116, 04347, Leipzig, Germany

**Email corresponding author: sontowski@igzev.de*

Additional experimental details, materials, methods, data processing, figures of experimental design, plant and insect performance, metabolites and volatiles

S1: Plant growth and insect-rearing conditions

OSR seeds were germinated in a 4 cm diameter plastic box filled with moistened vermiculite in a climate chamber (Percival, CLF plant climatics, Wertingen, Germany) at a constant temperature of 20°C and a ratio of 16:8 hours of light:darkness. The seeds were watered twice a week. After 14 days, the seedlings were transferred to the greenhouse and planted in 1-liter pots with moistened sand. They were grown at 22 - 31°C, a relative humidity of 30 - 60 % and 310 - 370 µmol/$m^2$s light in a 16:8h light:darkness ratio. The plants were watered as required and fertilized twice a week with 0.5 % Wuxal Super (Hauert Manna). These growth parameters were used in all experiments.

The insect *D. radicum* originated from a laboratory culture at the German Centre for Integrative Biodiversity Research (iDiv) in Leipzig, Germany. The colony was kept under controlled environmental conditions at a constant 22°C and a day:night rhythm of 16 to 8 h. We collected larvae and adult females from these cultures for all subsequent experiments.

S2: Root metabolic profile of selected resistant and susceptible accessions

Chromatographic separations were performed at 40°C on an UltiMate™ 3000 Standard Ultra-High Pressure Liquid Chromatography system (UHPLC, Thermo Scientific) equipped with an Acclaim® Rapid Separation Liquid Chromatography (RSLC) 120 column (150 × 2.1 mm, particle size 2.2 µm, ThermoFischer Scientific) with a flow rate of 0.4 ml/min and the following gradient: at 0-1 min, isocratic 95 % A (water/formic acid 99.95/0.05 (v/v %)) and 5 % B (acetonitrile/formic acid 99.95/0.05 (v/v %); at 1-2 min, accessionar from 5 to 20 % B; at 3-8 min, accessionar from 20 to 25 % B; at 8-16 min, accessionar from 25 to 95 % B; at 16-18 min, isocratic 95 % B; at 18 -18.01 min, accessionar from 95 to 5 % B; at 18.01-20 min, isocratic 5 % B. The injection volume was 1 µL (full loop injection).

Eluted compounds were detected from m/z 90 to 1600 at a spectra rate of 5 Hz (accession spectra only), using an ESI-UHR-Q-ToF-MS (maXis impact, Bruker Daltonics) equipped with an Apollo II electrospray ion source in negative and positive ion mode. In the negative mode, the following device settings were used: Nitrogen as a nebulizer gas at 2.5 bar and as dry gas at 11 L/min at 220°C. The capillary voltage was set to 4000 V, the end plate offset to 500 V, the radio frequency (RF) of funnel 1 to 200 volts peak-to-peak (Vpp), and the RF of funnel 2 to 220 Vpp. Furthermore, the in-source

collision-induced dissociation (CID) energy was set to 0.0 eV, hexapole RF to 120 Vpp, the quadrupole ion energy to 5 eV and the quadrupole low mass to 100m/z. Nitrogen was used as collision gas with a collision energy of 10 eV and set the prepulse storage to 8 µs. Stepping was switched on in Basic mode. The RF of the collision cell ranged from 400 Vpp to 1000 Vpp. The transfer time was set from 30 µs to 70 µs, the timing to 50 %/50 %, and collision energy for MSMS to 80 %. For positive ion mode, the same setup was used as for the negative mode, except that the capillary voltage was set to 4500 V, the quadrupole ion energy to 4 eV and a prepulse storage of 7 µs.

Calibration of the m/z scale was performed for single raw data files of sodium formate cluster ions prepared by automatic infusion of 1.66 µL/min of 10 mM sodium format solution of NaOH in 50/50 (v/v %) isopropanol water containing 0.2% formic acid at the end of the gradient (HPC mode).

For the acquisition of CID (collision-induced dissociation) mass spectra, the same settings as above were used with additional settings for data-dependent acquisition (AutoMSMS). Mode: CID, intensity threshold 600, number of precursors 5, precursor background subtraction on, active exclusion on after two spectra, release after 0.5 min, Smart exclusion on (5x), isolation and fragmentation settings, size- and charge-dependent, width 3-15 m/z, collision energy 20-30 eV, charge states included 1z, 2z and 3z.

The LC-MS data measured in the positive and negative modes were processed separately using MZmine version 3.9.0 (Schmid et al., 2023). The raw data were imported, and the mass was determined from the MS1 and MS2 data. A chromatogram was created using the ADAP Chromatogram builder, chromatograms were smoothed, C13 isotopes were filtered, isotope peaks were searched, and an alignment was created using the Join Aligner algorithm. Features of blanks were subtracted, duplicated peaks were removed, and an ion identity network was performed. Annotated compounds were matched against an in-house library of analytical standards of plant metabolites, the NIST17, and GNPS (https://gnps.ucsd.edu) based on mass, retention time, spectrum and spectral similarity. Furthermore, molecular formulas were identified, and compound classes were predicted from the features using Sirius version 5.8.3 (Djoumbou Feunang et al., 2016; Dührkop et al., 2019; Dührkop et al., 2021; Dührkop et al., 2015; Kim et al., 2021; Ludwig et al., 2019).

S3: Wax layer metabolome

The derivatized compounds were separated on a SH-Rxi-5ms column (30m x 0.25mm x 0.25µm, Shimadzu). Of the derivatized extract, 1 µl was injected with a split ratio of 10:1 and an injection temperature of 250°C. Helium was used as carrier gas with a column flow rate of 1.1 ml/min and a constant accessionar velocity of 36 cm/s. The column oven was held at 50°C for 2 min and then ramped

to 225°C at a rate of 35°C/min, held for 1 min and then ramped to 320°C at a rate of 3°C/min and finally held for 5 min. Electro impact (EI) spectra were recorded in scan mode from 35 to 650 m/z at a scan speed of 5000 Da/s from 3 to 60 min. The transfer accession was held at 290°C and the ion source at 250°C. Data processing was performed using Shimadzu GCMS Solutions software (version 4.52). We selected the 140 most conspicuous peaks in the chromatograms. Features contained in blank samples were excluded from the following processes. In addition, the features were manually screened for contaminations and removed. The remaining features were tentatively identified by comparing the spectra with the NIST20 database (National Institute of Standards and Technology, Gaithersburg, MD, USA) and Kovats retention indices with the literature. The peak area was calculated using GCMS Solutions software (version 4.52). The peak area was normalized to the area of the internal standard to compensate for inaccuracies during extraction. In addition, the peak area was normalized by estimating the leaf size (leaf length x leaf width) divided by leaf weight.

S4: VOC profiling

VOCs were desorbed under a 60 ml/min nitrogen flow for 8 min at 230°C. All desorbed substances were cryofocused at -20°C and desorbed at 250°C for 3 min. VOCs were injected splitless and helium was used as carrier gas at a constant velocity of 38 cm/s. The column oven was held at 45°C for 5 min. Afterward, the temperature ramped to 250°C at 10°C/min and finally held for 2 min. Electro impact (EI) spectra were acquired in scan mode from 35 to 450 m/z at a scan speed of 5000 Da/s. The transfer accession was held at 230°C and the ion source at 230°C. Data processing was performed in Shimadzu GCMS solutions software (version 4.52). We selected the 80 most prominent peaks in the chromatograms. Compounds measured in empty PDMS tubes and empty pots were excluded from the following processes. The remaining compounds were tentatively identified by comparing the spectra with the NIST20 database (National Institute of Standards and Technology, Gaithersburg, MD, USA) and the Kovats retention indices from the literature. In addition, the compounds were manually checked for contaminations and removed. Hits were compared to the LOTUS natural product database (42) to support their organic origin. The peak area was calculated using GCMS Solutions software (version 4.52).

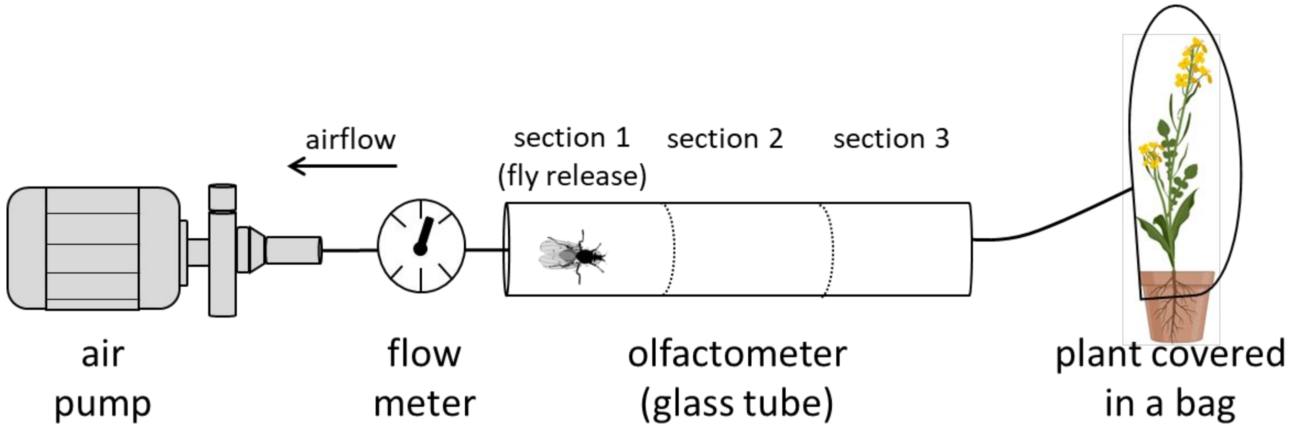


Figure S1: Schematic setup of the linear olfactometer used for the no-choice attractiveness assay to test for female preference based on plant volatiles. Female flies were placed in section 1 at the start point of the experiment, which was the furthest point from the plant. The olfactometer was divided into three equal sections (1–3). The female flies were observed for 10 minutes. The time they spent in sections 1, 2 and 3 was monitored. The time spent in section 3 (the section closest to the plant) was used as an indicator of attraction to the plant. Time spent in section 1 (close to the release point) was associated with a lack of response or unattractiveness of the plant.

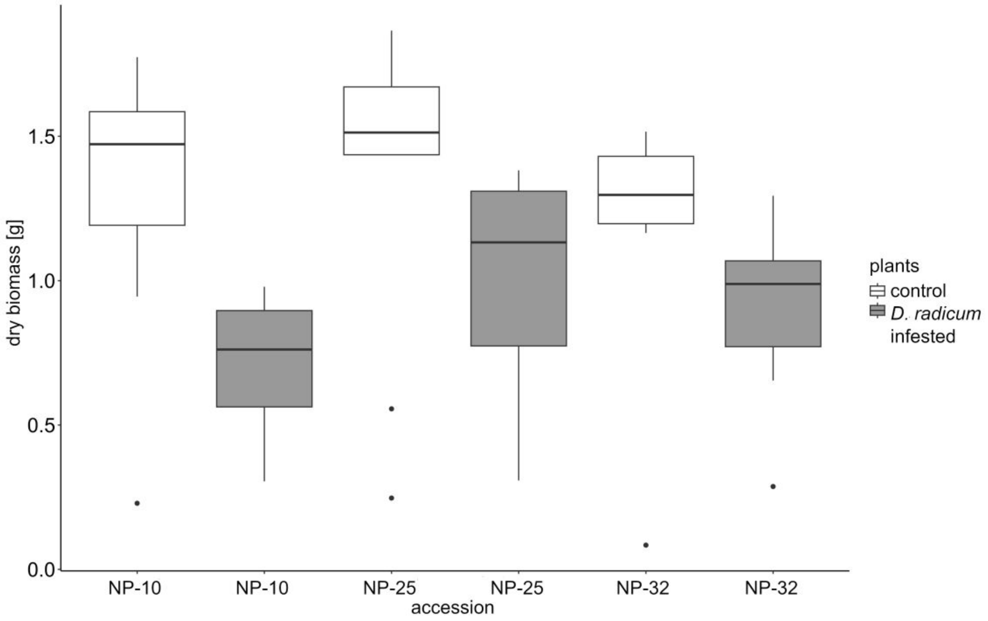


Figure S2: Boxplot of root dry mass of *B. napus* accessions from control (white) and *D. radicum*-infested plants (grey). Only plant accessions with significant differences are presented in the figure.

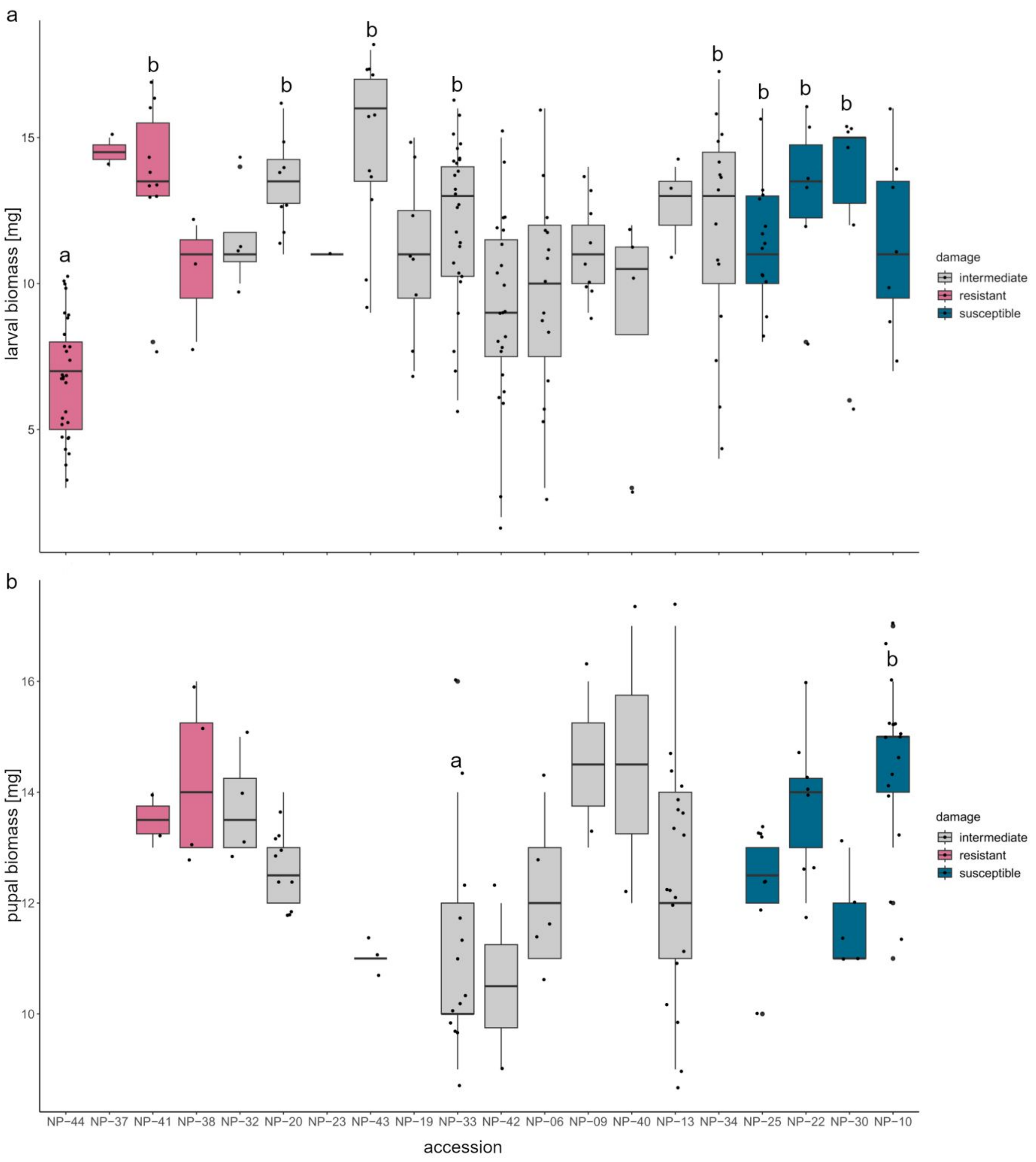


Figure S3: Insect performance represented by larval and pupal biomass of *D. radicum*. Boxplots represent the biomass of *Delia radicum* larvae (a) and pupae (b), feeding on 20 *B. napus* accessions. Colors indicate plant susceptibility categories to *D. radicum* larvae based on damage rankings in the greenhouse experiment; resistant (magenta), susceptible (blue) and medium (grey). Different letters indicate significant differences in body mass with an adjusted $P < 0.05$ (Bonferroni) based on Kruskal-Wallis test followed by Dunn Test.

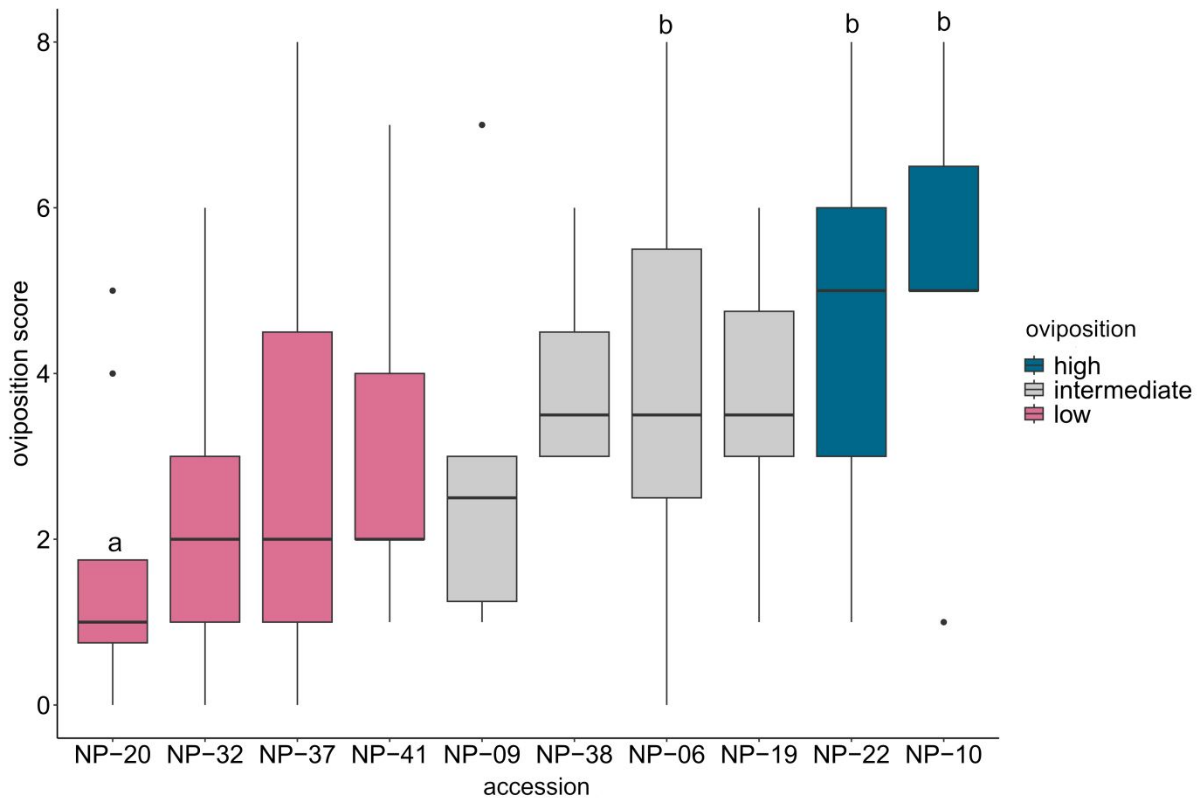


Figure S4: Oviposition score of *D. radicum* on 10 *B. napus* accessions in a series of choice experiments. Colors indicate oviposition categories of *D. radicum* females. Oviposition score includes the number of eggs laid and the preferred accession amongst four plants, whereby 0 indicates the lowest preference category and 8 the highest. The letters indicate P < 0.05 based on a GLM model with quasi-poisson distribution using accession NP-20 as a reference.

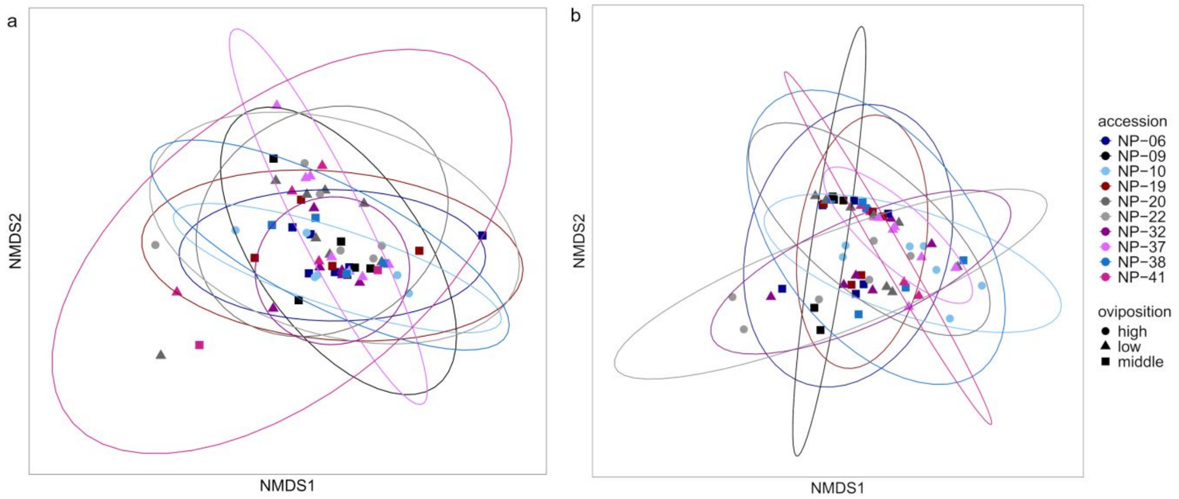


Figure S5: NMDS plots of distances based on the peak area of 10 *B. napus* accessions. (a) NMDS plot of plant volatile organic compound (VOC) features produced by 10 plant accessions, collected on PDMS tubes in 24h from whole plants using passive trapping. (b) NMDS plot of cuticular wax layer metabolome of 10 *B. napus* accessions extracted from the youngest fully developed leaf. Each dot represents one plant, the different colors represent different accessions and the shape the

oviposition class of *D. radicum* (circle – high oviposition rate, triangle – low oviposition rate, square – middle oviposition rate).

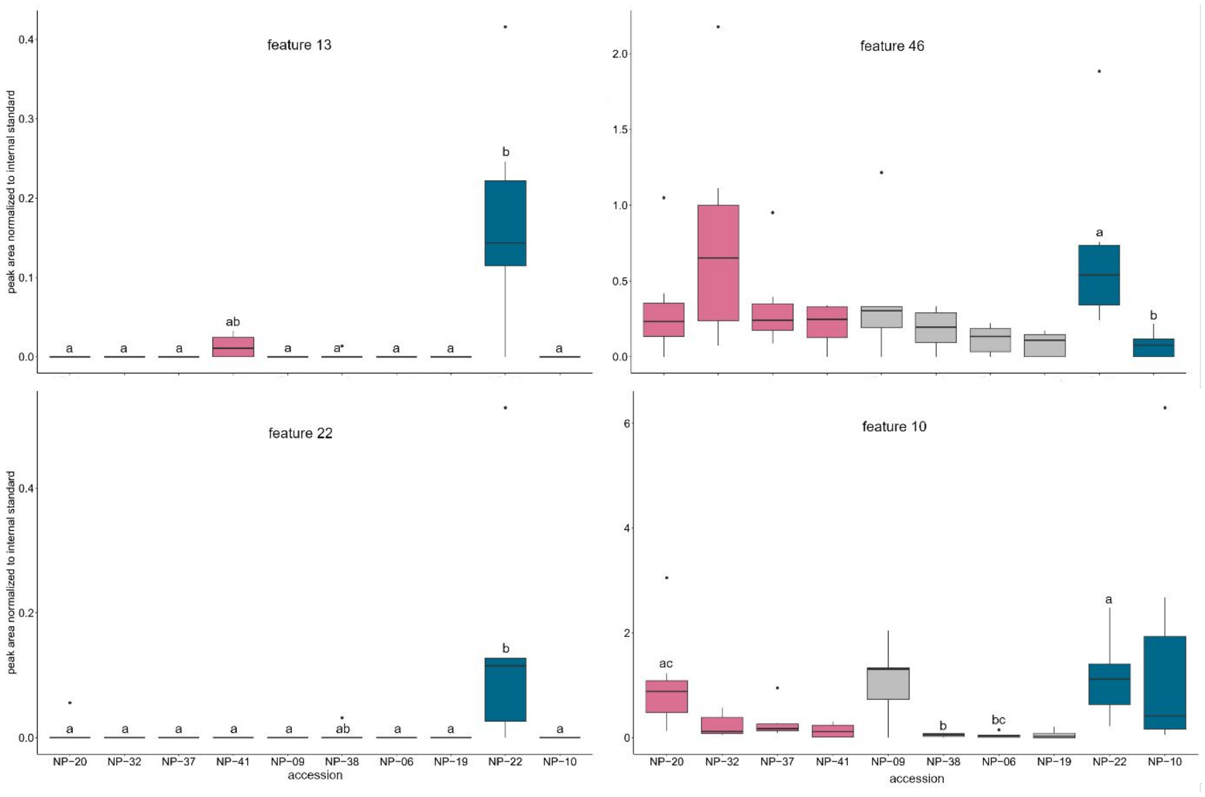


Figure S6: Boxplot of peak area normalized to internal standard from selected cuticular wax layer metabolic features measured with GC-MS in 10 *B. napus* accessions. Features were selected based on the differences between accessions. Red color indicates accessions with low oviposition rate by *D. radicum* females, blue color indicates preferred accessions by *D. radicum* females for oviposition and grey color indicates a middle oviposition rate. Letters indicate significant differences ($P < 0.05$) after *Kruskal-Wallis* followed by *Dunn's Test* with *Bonferroni*-adjusted P-values.

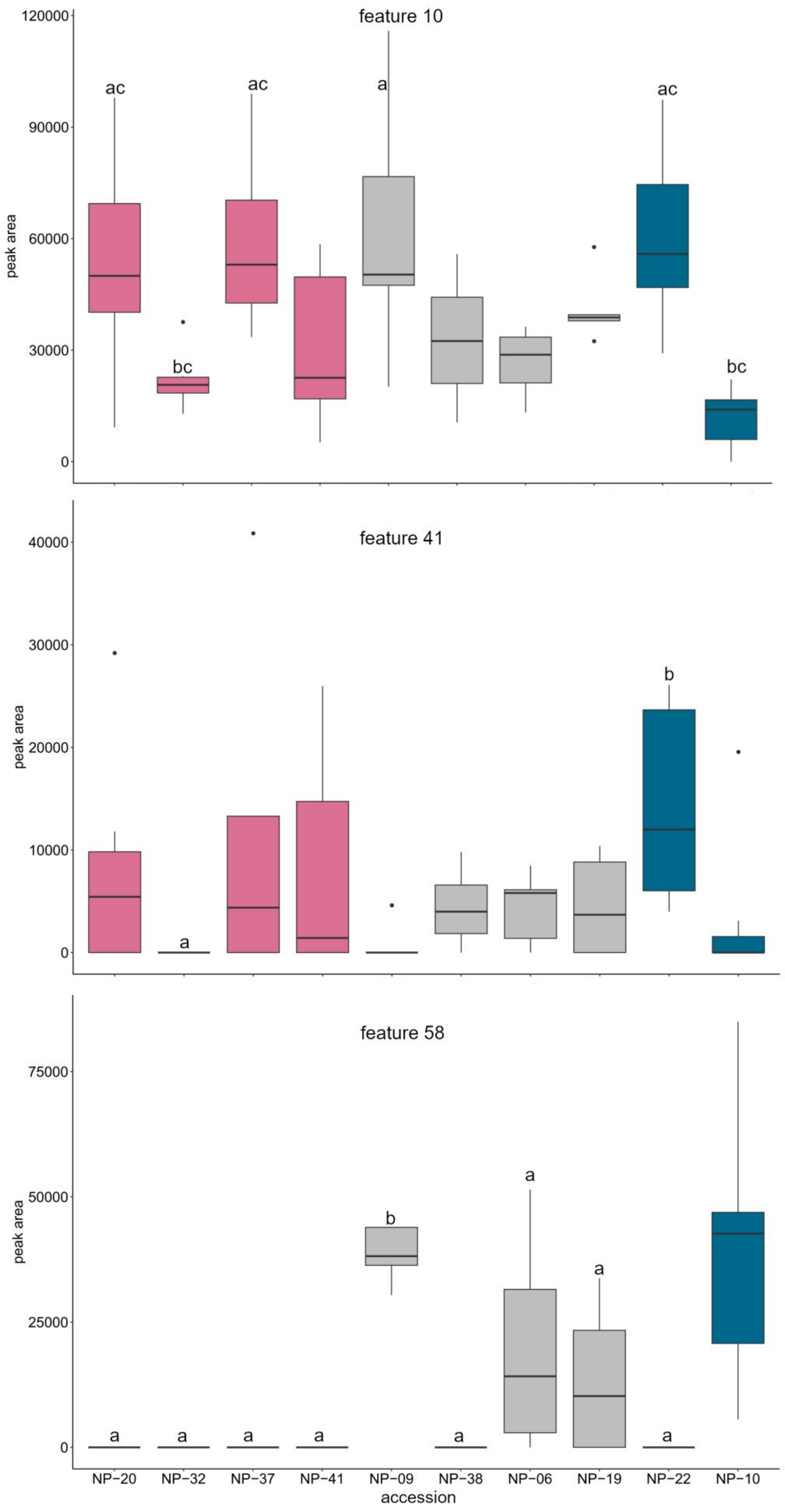


Figure S7: Peak area of three selected plant volatile organic compound features in 10 *B. napus* accessions collected with PDMS tubes after 24h passive trapping. Features were selected based on significant differences among plant accessions. Red color indicates accessions with low oviposition

rate by *D. radicum* females, blue color indicates preferred accessions by *D. radicum* females for oviposition and grey color indicates accessions with a middle oviposition rate. Letters indicate significant differences ($P < 0.05$) after one-way *ANOVA* followed by *Tukey HSD*.
